# Evolved strains of *Scheffersomyces stipitis* achieving high ethanol productivity on acid- and base-pretreated biomass hydrolyzate at high solids loading

**DOI:** 10.1186/s13068-015-0239-6

**Published:** 2015-04-09

**Authors:** Patricia J Slininger, Maureen A Shea-Andersh, Stephanie R Thompson, Bruce S Dien, Cletus P Kurtzman, Venkatesh Balan, Leonardo da Costa Sousa, Nirmal Uppugundla, Bruce E Dale, Michael A Cotta

**Affiliations:** Bioenergy Research Unit, National Center for Agricultural Utilization Research, USDA-ARS, 1815 N. University, Peoria, IL 61604 USA; Bacterial Foodborne Pathogens and Mycology Research, National Center for Agricultural Utilization Research, USDA-ARS, 1815 N. University, Peoria, IL 61604 USA; DOE Great Lakes Bioenergy Research Center, Michigan State University, East Lansing, MI 48824 USA

**Keywords:** Lignocellulose, Biofuel, Adaptation, Yeast, *Pichia stipitis*, Fermentation

## Abstract

**Background:**

Lignocellulosic biomass is an abundant, renewable feedstock useful for the production of fuel-grade ethanol via the processing steps of pretreatment, enzyme hydrolysis, and microbial fermentation. Traditional industrial yeasts do not ferment xylose and are not able to grow, survive, or ferment in concentrated hydrolyzates that contain enough sugar to support economical ethanol recovery since they are laden with toxic byproducts generated during pretreatment.

**Results:**

Repetitive culturing in two types of concentrated hydrolyzates was applied along with ethanol-challenged xylose-fed continuous culture to force targeted evolution of the native pentose fermenting yeast *Scheffersomyces* (*Pichia*) *stipitis* strain NRRL Y-7124 maintained in the ARS Culture Collection, Peoria, IL. Isolates collected from various enriched populations were screened and ranked based on relative xylose uptake rate and ethanol yield. Ranking on hydrolyzates with and without nutritional supplementation was used to identify those isolates with best performance across diverse conditions.

**Conclusions:**

Robust *S. stipitis* strains adapted to perform very well in enzyme hydrolyzates of high solids loading ammonia fiber expansion-pretreated corn stover (18% weight per volume solids) and dilute sulfuric acid-pretreated switchgrass (20% w/v solids) were obtained. Improved features include reduced initial lag phase preceding growth, significantly enhanced fermentation rates, improved ethanol tolerance and yield, reduced diauxic lag during glucose-xylose transition, and ability to accumulate >40 g/L ethanol in <167 h when fermenting hydrolyzate at low initial cell density of 0.5 absorbance units and pH 5 to 6.

## Background

An estimated 1.3 billion dry tons of lignocellulosic biomass could be available annually to support ethanol production at a level that would allow the U.S. to reduce its petroleum consumption by 30% [1]. The fibrous, plant cell-wall material that is characteristic of lignocellulosic feedstocks is highly recalcitrant and difficult to deconstruct into fermentable sugars. The chemical pretreatment required to open the structure of plant biomass to enzymatic hydrolysis results in solutions rich in glucose and xylose. However, pretreatment generates fermentation-inhibiting byproducts, including acetic acid, furfural, hydroxymethyl furfural (HMF), and others. Traditional industrial yeasts do not ferment xylose and are not able to survive, grow, or ferment in toxic concentrated hydrolyzates which contain high sugar concentrations (>100 g/L) to support ethanol accumulation (>40 g/L) high enough for economical recovery [2,3].

*Pichia stipitis* is known to ferment D-xylose to ethanol more efficiently than other native yeasts previously described [4]. The species, represented by type strain NRRL Y-7124 (CBS 5773), was recently renamed *Scheffersomyces stipitis* [5] and is particularly useful because it has strong NADH-linked as opposed to NADPH-linked aldose reductase activity providing for a more favorable cofactor balance in the conversion of xylose to xylulose [6] and a high ethanol yield. Strain NRRL Y-7124 was selected for our adaptation study because it ferments hexoses and xylose to economically recoverable concentrations of ethanol exceeding 40 g/L with almost no accumulation of xylitol byproduct [7-9]. In nutritionally optimized media, *S. stipitis* strain NRRL Y-7124 is able to produce over 70 g/L ethanol in 40 h (1.75 g/L/h) from 150 g/L sugars at a yield of 0.41 ± 0.06 g/g in high density fermentations (6 g/L cells) [7,10,11]. Given appropriate nitrogen levels and sources, it is also relatively resistant to fermentation inhibitors ethanol, furfural, and HMF [12]. *S. stipitis* is one of the most viable native pentose-fermenting yeasts available for commercial scale-up [13]. For industrial application, sugar uptake rate in biomass hydrolyzates needs improvement, including reducing the effects of diauxy and improving ethanol and inhibitor tolerance. To advance the science and application of *S. stipitis,* our objective was to apply appropriate selective pressure to guide its evolution toward an industrially robust derivative that is tolerant of diverse lignocellulosic hydrolyzates.

Two different types of industrially promising hydrolyzate were selected for application in our adaptation process, namely ammonia fiber expansion-pretreated corn stover hydrolyzate (AFEX CSH) [14,15] and enzymatically saccharified dilute acid-pretreated post-frost switchgrass (SGH). In contrast to AFEX CSH, SGH is characterized by high contents of furan aldehydes and acetic acid and very low levels of available nitrogen needed to support yeast growth and fermentation. Dilute acid-pretreated switchgrass hydrolyzate liquor (PSGHL) is the liquor in association with the hydrolyzed solids. It is separable from the solids by filtration or centrifugation and is characteristically rich in xylose but low in glucose, a feature which may make PSGHL a useful enrichment medium to force selection for improved xylose utilization in hydrolyzates, a failing point for many types of native and engineered yeast tried in the past. The low available N content of the switchgrass hydrolyzates provided opportunity to explore the utility of nitrogen supplementation in the process of screening and ranking improved strains. Both AFEX CSH and PSGHL were used as challenging selective media applied in sequence and in parallel to force the evolution of *S. stipitis* toward derivatives with enhanced ability to grow and ferment in diverse hydrolyzates. The repetitive culturing and retrieval of functional populations from increasingly concentrated hydrolyzate environments was the general strategy to be accomplished in microplates employing a dilution series of 12% glucan AFEX CSH or PGSHL prepared at 20% solids loading. This strategy utilized natural selection and enrichment to recover spontaneous hydrolyzate inhibitor-tolerant derivatives of strain NRRL Y-7124.

An additional feature of this research was the application of ethanol-challenged continuous culture to further enhance and stabilize AFEX CSH-adapted populations. Ethanol-challenged xylose growth and fermentation was targeted to enrich for populations able to resist ethanol toxicity, grow, and survive on xylose as a sole C source and able to induce xylose-specific enzymes allowing fermentation of xylose to ethanol, even in the presence of high concentrations of ethanol. Recent investigations [11] have shown that ethanol concentrations around 15 to 50 g/L progressively repressed enzyme inductions specific to xylose utilization. The resulting populations enriched in ethanol-tolerant derivatives of the AFEX CSH tolerant population were next subjected to further enrichment on PSGHL to explore this approach to broadening the functionality of strains in various types of hydrolyzates.

Once adaptations were completed, isolates were evaluated. Optimally adapted individuals from populations occurring at various phases of adaptation were obtained by enrichment under target stress conditions followed by dilution plating to skim the most prevalent populations from which to pick colonists. Selected colonists were then ranked using dimensionless relative performance indices to determine best overall performance considering xylose uptake rate and ethanol yield on various hydrolyzates with or without nutrient supplementations. To improve the performance of *Scheffersomyces* strains, several researchers have applied various adaptation procedures [16-20]. Although the potential utility of adaptation to improving the functionality of *Scheffersomyces* strains in lignocellulosic hydrolyzates is suggested by the literature, application of adapted strains of *S. stipitis* to hydrolyzates with economical ethanol production has not been reported. The evolved strains reported in this paper are significantly improved over the parent NRRL Y-7124 in accordance with high solids loading hydrolyzate screening targets and are able to produce >40 g/L ethanol in AFEX CSH and in appropriately soy nitrogen-supplemented SGH. Given the sequenced genome of a second *S. stipitis* strain, NRRL Y-11545 [21], these novel strains are candidates for future studies to uncover the genetic changes that have occurred and the mechanisms underlying hydrolyzate tolerance and improved fermentation.

## Results and discussion

**Figure 1:**
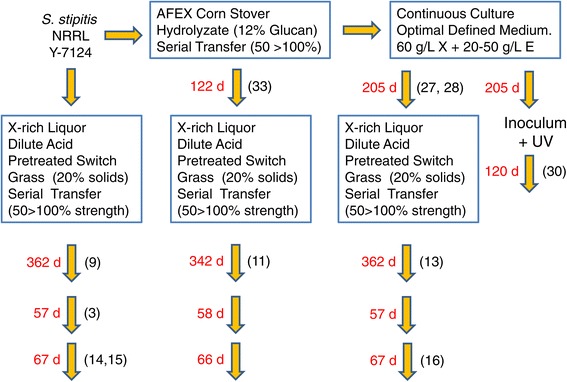
*Scheffersomyces stipitis* adaptation flow chart. The diagram shown indicates the order of the stresses applied during the adaptation process and the points of recovery of superior isolates (numbers in parenthesis). See also Table 2 isolate key as reference for strain identities. To provide time orientation, the numbers in red indicate the number of days in each phase of adaptation. For the serial transfer phases in AFEX CSH and xylose-rich PSGHL, each day of adaptation represents approximately two to four generations. For the continuous culture phase (205 days total), the dilution rate D was variable at approximately 0 to 0.1 h^−1^ during 125 days of operation with pH-actuated feeding. In the next 80 days, operation was at a continuous flow with D at 0.012 h^−1^, providing a generation time (ln 2/D) of 58 h or one generation per 2.4 days at steady state. Next, a sample of the adapted population from the 205-day continuous culture was mutagenized with UV light and inoculated to a continuous culture operated with D at 0.012 h^−1^.

Figure 1 provides a flow diagram of the adaptation processes used to obtain the tolerant strains reported and indicates the screening numbers and points of isolation for superior ranking strains characterized either by consistently high xylose consumption rates and high ethanol yields across all hydrolyzate formulations (Table 1) or by extremely high, consistent performance on at least one type of hydrolyzate. On the basis of identical nucleotide sequences for domains D1/D2 of the nuclear large subunit rRNA gene, all superior tolerant isolates were identified as *S. stipitis* [22] and deposited in the ARS Patent Culture Collection with the accession numbers listed in Table 2 along with adaptation stresses applied during strain evolution and screening numbers used in graphs. Results will be noted and discussed in order of the adaptation sequence.

**Table 1:** **Compositions of hydrolyzates used in cultivations** ^**a**^

**Component** ^**b**^	**AFEX-pretreated corn stover hydrolyzate**				**Dilute acid-pretreated switch grass hydrolyzates (20% solids)**							
	**6% Glucan**		**12% Glucan**		**PSGHL**		**SGH**		**SGH-N1**		**SGH-N2**	
	**Mean**	**s**	**Mean**	**s**	**Mean**	**s**	**Mean**	**s**	**Mean**	**s**	**Mean**	**s**
Glucose (g/L)	58.9	9.0	107.4	16.7	7.9	2.5	69.2	3.2	67.4	6.5	64.2	1.3
Xylose (g/L)	34.2	7.3	48.7	15.8	52.1	5.1	48.6	2.7	45.3	3.5	47.4	0.8
Arabinose (g/L)	4.3	0.6	9.5	0.4	7.6	0.9	6.1	0.3	4.4	0.7	7.6	0.2
Galactose (g/L)	3.1	0.3	5.7	0.6	3.0	0.7	5.2	0.3	5.2	0.3	5.2	0.3
Fructose (g/L)	4.0	1.7	8.2	3.0	0.9	0.4	0.7	0	0.7	0	0.7	0
Mannose (g/L)	1.0	0.1	2.0	0.2	8.9	5.1	0	0	0	0	0	0
Acetic acid (g/L)	1.8	0.2	4.7	1.0	6.1	2.8	5.4	0.4	4.3	0.6	5.8	0.3
HMF (mM)	0.3	0.5	1.1	1.5	2.8	3.1	1.8	0.2	1.1	1.3	3.6	0.5
Furfural (mM)	0.2	0.1	0.4	0.0	24.4	7.6	18.3	4.5	24.5	4.5	19.1	0.8
PAN (mg N/L)	318.7	49.9	493.2	69.1	33.7	15.6	69.9	14.7	188.0	4.0	173.0	22.0
Urea (mg N/L)	83.1	29.3	105.5	9.1	0.7	0.7	7.0	2.7	1101.0	--^c^	962.0	105.0
Ammonia (mg N/L)	1,193.4	289.7	2,707.6	385.6	25.0	21.0	23.0	24.6			369.0	63.0

^a^Values are reported in terms of mean and standard deviation(s) across hydrolyzates used in experiments reported.

^b^Abbreviations: HMF = hydroxymethylfurfural; PAN = primary amino nitrogen; PSGHL = dilute acid pretreated switchgrass hydrolyzate liquor; SGH = switchgrass hydrolyzate; or with nutrient supplements -N1 or -N2, as described in ‘Methods’ section.

^c^No values of *s* since N content was calculated based on urea addition.

**Table 2:** **Summary of superior tolerant** ***Scheffersomyces stipitis*** **strains for fermentation of hydrolyzates of plant biomass**

**Accession number (NRRL)**	**Screening number**	**Isolate designation**	**Adaptation stress** ^**a**^	**Preferred hydrolyzate** ^**a**^
Y-50871	33	Colony 5	AFEX CSH	All; AFEX CSH
Y-50872		Colony 1	AFEX CSH	NR
Y-50873		Colony 7	AFEX CSH	NR
Y-50861	27	2A.1.53R-E20-C1	AFEX CSH > E	All; AFEX CSH
Y-50862	28	2A.1.53R-E30-C3	AFEX CSH > E	All; SGH-N2
Y-50864	30	2A.30R2-E40-C5	AFEX CSH > E (UV)	SGH-N2; AFEX CSH
Y-50857	13	2A.1.53R S100E40-1	AFEX CSH > E > PSGHL	All
Y-50860	16	2A.1.53R-1	AFEX CSH > E > PSGHL	SGH-N1 or -N2
Y-50865	11	Colony 5 GP-6	AFEX CSH > PSGHL	SGH-N2 or -N1
Y-50874	3	Y-7124 S90E40-1	PSGHL	All
Y-50863	9	Y-7124 GP-5	PSGHL	SGH-N1 or -N2
Y-50859	14	Y-7124-6	PSGHL	All
Y-50858	15	Y-7124-10	PSGHL	All

^a^Abbreviations are as follows: AFEX CSH = ammonia fiber explosion-pretreated corn stover enzyme hydrolyzate; E = ethanol-fed continuous culture; UV = ultraviolet light-treated inocula for E; PSGHL = dilute acid-pretreated switchgrass hydrolyzate liquor; NR = not ranked.

### Adaptation to enzyme saccharified AFEX CSH in microplate batch cultures

**Figure 2:**
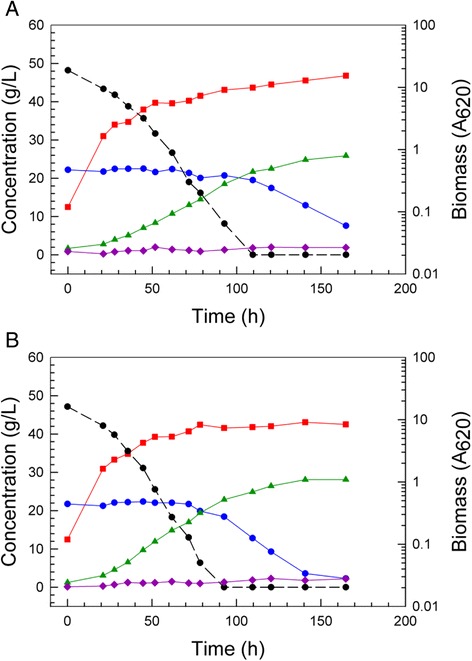
Improved batch fermentation of 6% glucan AFEX CSH. *Scheffersomyces stipitis* NRRL Y-7124 parent strain fermentation of 6% glucan AFEX CSH (**A**) is compared with adapted colony 5 fermentation of 6% glucan AFEX-pretreated corn stover hydrolyzate (**B**). Symbols designate biomass (red square), glucose (black circle with dashed line), xylose (blue circle with solid line), ethanol (green triangle), and xylitol (purple diamond).

After the parent strain NRRL Y-7124 was exposed to increasing concentrations of 12% glucan AFEX CSH for several weeks, an adapted population was stored as a glycerol stock, and when a single colony isolate (colony 5) was cultivated on 6% glucan AFEX CSH in comparison with the parent strain, a significantly enhanced performance was observed as shown in Figure 2. Superior performance features of the adapted population isolate, colony 5, included faster glucose and xylose volumetric uptake rates (that is, 27% and 67% increases, respectively), more complete xylose uptake (2 g/L *versus* 7 g/L), and higher ethanol production rate (16% increase) and accumulation (7.7% increase).

**Figure 3:**
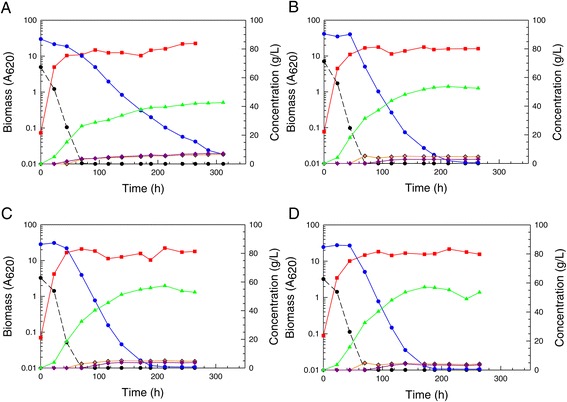
Reduced diauxic lag in defined medium with mixed sugars. Fermentation performances are compared in ODM with 66 g/L glucose and 87 g/L xylose for parent strain *S. stipitis* NRRL Y-7124 (**A**), the AFEX CSH adapted population derived from Y-7124 (**B**), single-cell colony 1 isolated from the adapted *S. stipitis* population (**C**), single-cell colony 5 isolated from the adapted population (**D**). Symbols designate biomass (red square), glucose (black circle with dashed line), xylose (blue circle with solid line), ethanol (green triangle), xylitol (purple diamond), and adonitol (gold diamond with black edge).

When single colony isolates of the adapted population were obtained and compared on optimal defined medium (ODM) with 66 g/L glucose and 87 g/L xylose against the parent NRRL Y-7124, all isolates showed similar performance to the adapted population and significantly less diauxic lag compared to the parent. Figure 3 shows relative performances and indicates that the adapted population and superior isolated single-cell clones (colonies 1, 5, and 7 (not shown)) were significantly improved in their ability to rapidly switch to xylose fermentation once glucose had been depleted. Both clones 1 and 5 shown had consumed all xylose by 200 h and made approximately 57 g/L ethanol, while the parent had 7 g/L xylose remaining even at 300 h and had accumulated only about 44 g/L ethanol. Prior research [11] has shown that the induction of enzymes specific for xylose metabolism is repressed in the parent NRRL Y-7124 when ethanol concentration exceeds 15 g/L. In the fermentations of Figure 3, ethanol reached nearly 30 g/L by the time glucose was depleted, and the adapted population and clones were not repressed in xylose utilization in contrast to the parent strain, which was severely crippled in its ability to use xylose after the glucose had been consumed. Exposure to increasing concentrations of AFEX-pretreated corn stover hydrolyzate, that is, increasing concentrations of sugars and inhibitors, led to an adapted population better able to ferment this hydrolyzate not only because of enrichment of the population in members more resistant to the inhibitory environment of the hydrolyzate but also because of enrichment of the population in members that were less susceptible to ethanol-associated repression of enzymes specific to xylose metabolism, thus avoiding extended diauxic lag.

**Figure 4:**
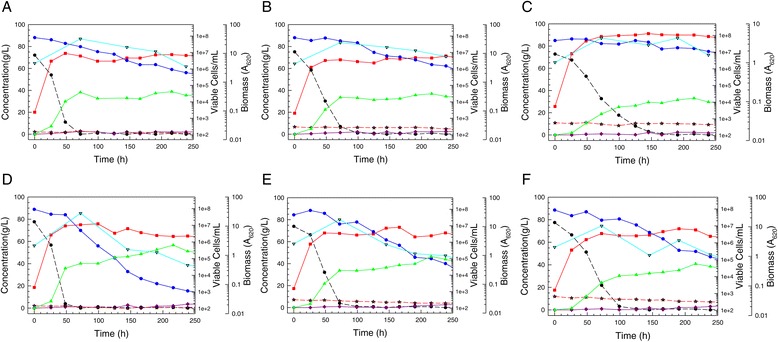
Reduced sensitivity to acetic acid in optimal defined medium. Sensitivity of *S. stipitis* NRRL Y-7124 in ODM to increasing acetic acid concentrations at 2 (**A**), 6 (**B**, and 10 g/L (**C**) is compared to relative tolerance of adapted colony 5 at the same acetic concentrations ((**D**, (**E**) and (**F**), respectively). The following time course data are shown: biomass (red squares), glucose (black circles and dashed line), xylose (blue circles and solid line), xylitol (purple diamonds), acetic acid (red stars), ethanol (green triangles), viable cells (inverted aqua triangles).

Acetic acid arises as a product of hemicellulose hydrolysis during pretreatment, and impaired abilities of engineered *S. cerevisiae* strains to shift from glucose to xylose utilization in the presence of 7.5 to 15 g/L acetic acid have been documented [23]. Consequently, the sensitivity of strain Y-7124 and its adapted derivatives to acetic acid was studied in ODM. After exposure to relatively low levels of acetic acid ( 2 to 4.7 g/L in dilutions of 12% glucan AFEX CSH) during the adaptation process, colony 5 was able to ferment both glucose and xylose in ODM containing 2 to 10 g/L acetic acid more efficiently than the parent strain NRRL Y-7124 (Figure 4), particularly due to reduced diauxic lag and more rapid xylose fermentation. Even at 10 g/L acetic acid, colony 5 continued to ferment xylose to ethanol, while the parent strain was not able to do so. Despite the strong impact of acetic acid on fermentation, the growth of both parent and colony 5 was relatively unimpaired across the concentrations used, although the numbers of viable colony forming units fell more in colony 5 than parent cultures, either due to greater cell flocculation or death or both.

**Figure 5:**
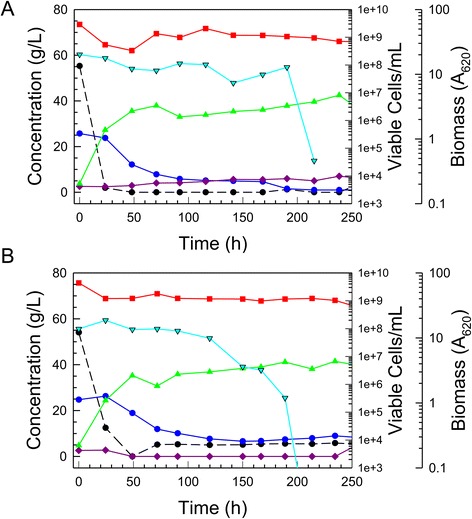
Fermentation of 8% glucan AFEX CSH to > 40 g/L ethanol with a high cell density of adapted strain. Fermentation batches (**A**) and (**B**) are shown for 8% glucan AFEX-pretreated corn stover hydrolyzate that was inoculated with a large population of *S. stipitis* AFEX CSH-tolerant colony 5 repitched from a 6% glucan batch growth during xylose utilization. Time courses of biomass (red squares), glucose (black circles and dashed line), xylose (blue circles and solid line), xylitol (purple diamonds), ethanol (green triangles), and viable cells (aqua inverted triangles) are shown.

**Figure 6:**
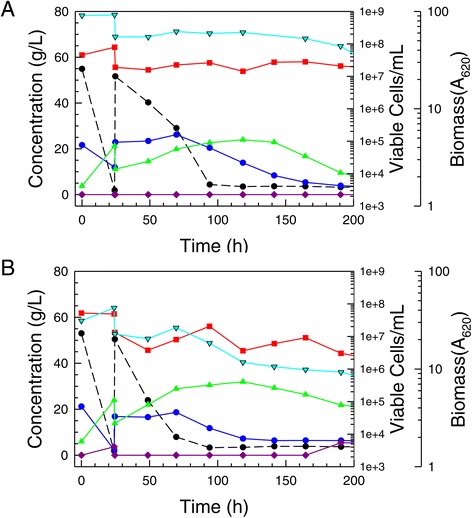
Comparative fed-batch fermentations. High density cultures of *S. stipitis* NRRL Y-7124 parent (**A**) and hydrolyzate-tolerant Colony 5 (**B**) repitched from 6% glucan during xylose uptake into fresh 6% glucan hydrolyzate were then fed at 24 h with an equal volume of 12% glucan hydrolyzate. Time courses of biomass (red squares), glucose (black circles and dashed line), xylose (blue circles and solid line), xylitol (purple diamonds), ethanol (green triangles), and viable cells (aqua inverted triangles) are shown.

To validate the application of colony 5, we applied high cell densities for rapid ethanol production from AFEX CSH. Replicate colony 5 populations grown on 6% glucan AFEX CSH and repitched to A_620_ of 40 in higher glucan hydrolyzates were tested and found to allow the accumulation of over 40 g/L ethanol on circa 8% glucan hydrolyzates (Figure 5). Although full accumulation of the ethanol required over 188 h, around 85% of it was accumulated in the first 48 h. Further testing of the adapted strain in comparison to its parent in populations repitched to A_620_ of 40 in 6% glucan hydrolyzate and later fed with an equal volume of 12% glucan hydrolyzate indicated a significant improvement in the adapted strain over the parent with respect to xylose uptake rate, ethanol productivity, and maximum ethanol accumulation (Figure 6) during both the initial batch and fed-batch phases of the cultivation. The adapted isolate was able to consume both glucose and xylose more quickly than the parent strain, reducing the sugar consumption time by 25% from 200 h to just under 150 h. In addition, ethanol accumulation of the adapted strain was improved by about 30% over that of the parent. However, xylose utilization was not complete and the adapted strain cell viability fell near the end of glucose consumption/beginning of xylose uptake, suggesting a potential problem combating deleterious impacts of ethanol and inhibitors from the 12% glucan AFEX CSH feed while trying to metabolize the xylose. It is notable that when compared to simple batch operation, the fed-batch process ethanol productivity and ethanol accumulation of colony 5 were lower by 30% and 22%, respectively, based on final volume, even though the fed-batch process was fed more sugar overall, 105 g/L compared to 80 g/L. Thus, the fed-batch mode of operation did not overcome the inhibitor challenges accompanying the abrupt addition of an equal volume of concentrated 12% glucose AFEX CSH hydrolyzate to the first stage 6% glucan AFEX CSH.

### Application of continuous culture to improve xylose utilization with increasing ethanol

**Figure 7:**
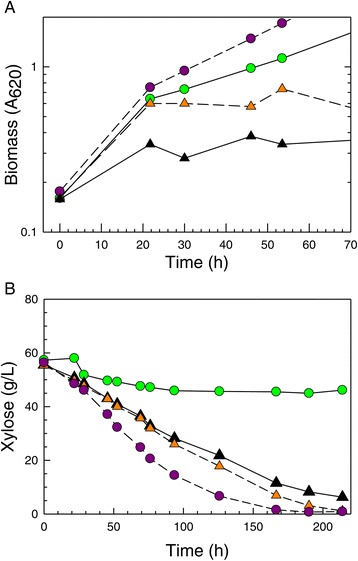
Ethanol-resistant derivatives of colony 5. Hydrolyzate-tolerant colony 5 was further developed by continuous culture selection on ODM containing xylose as sole carbon source and high levels of ethanol. (**A**) The growth rate on ODM + 60 g/L xylose +40 g/L ethanol of two derivative glycerol stock populations obtained early in the selection process (2A.1.53R, orange triangle and dashed line) and after UV irradiation of continuous culture inocula (2A.1.30R.2, purple circle and dashed line) are shown in comparison with the NRRL Y-7124 parent strain (green circle with solid line) and AFEX CSH-tolerant colony 5 (black triangle with solid line). (**B**) Xylose uptake by dense populations of glucose-grown yeast (A_620_ = 50) in ODM with 40 g/L ethanol indicated that all adapted strains surpassed the unadapted parent in the ability to induce xylose metabolism.

To improve ethanol tolerance and resistance to repression of enzymes specific to xylose utilization, colony 5 was inoculated to a continuous culture operated at a low dilution rate with high ethanol concentrations and xylose in the feed. The goal was to select and enrich for yeast cells with improved growth and fermentation on xylose as sole carbon source in the presence of high ethanol concentrations. Cells were exposed to >15 g/L ethanol, which is associated with repression of xylose-specific enzymes in the parent strain NRRL Y-7124 [11]. In Figure 7a,b, the progressive improvement in initial growth rate on xylose in the presence of 40 g/L ethanol is seen for two enriched populations, 2A.1.53R and 2A.1.30R2, which had been collected at earlier and later times, respectively, during operation and then frozen in glycerol. Population 2A.1.30R2 was obtained from the continuous culture following reinoculation with UV-mutagenized populations and further enrichment under 40 to 50 g/L ethanol challenge. These data suggest that the ethanol resistance loss shown by colony 5 was alleviated by the continuous culture process and UV exposure. In addition, the ability of glucose-induced cells to switch to xylose metabolism in the presence of 40 and 45 g/L ethanol was shown to be further improved over that of the colony 5 inoculum and far better than that of the parent control (Figure 7a,b). The enriched populations 2A.1.30R2 and 2A.1.53 R were successful at using xylose even in the presence of >40 g/L ethanol when yeast had been cultivated on glucose and transferred in high density to ODM with xylose and ethanol (Figure 7b). Three single-cell isolates (2A.1.53R-E20-C1, 2A.1.53R-E30-C3, and 2A.30R2.E40-C5) were recovered from these populations, which showed superior ability to ferment hydrolyzates in subsequent screens.

### Adaptation to PSGHL in microplate batch cultures

To further broaden hydrolyzate tolerance, colony 5 and the ethanol-tolerant derivative population 2A.1.53R were further challenged by repetitive serial transfer to increasing concentrations of xylose-rich PSGHL in microplates. Additionally, strain NRRL Y-7124 was subjected to direct adaptation in PSGHL as a control relative to sequential application of different adaptation challenges. Using dilution plating, over 150 isolates were recovered either directly from adaptation microplates or from frozen glycerol stock populations after streaking to YM agar and selective enrichment with hydrolyzate and/or ethanol challenges. Four superior hydrolyzate-fermenting isolates were identified from NRRL Y-7124 adaptation to PSGHL (Y-7124 GP-5, Y-7124 S90E40-1, Y-7124-6, and Y-7124-10) and one each was derived from colony 5 and population 2A.1.53R (colony 5 GP-6 and 2A.1.53R-1, respectively) when the following screening and ranking processes were applied (Table 2).

### Primary screen of isolates on xylose-rich PSGHL

**Figure 8:**
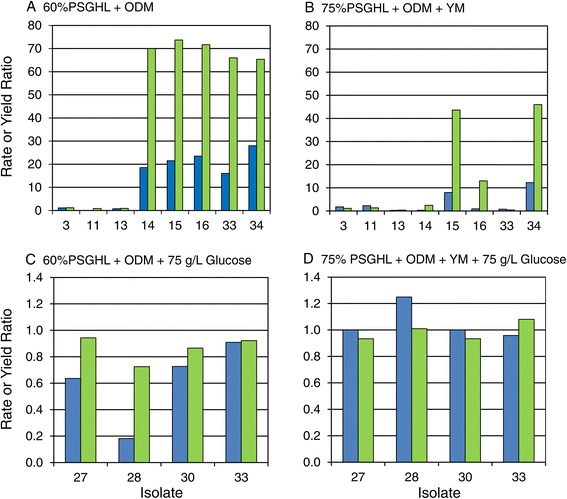
Ratio of performance improvement of tolerant isolate compared to parent. The performances of superior tolerant isolates are summarized relative to the control parent strain NRRL Y-7124 for each formulation of PSGHL (**A**-**D**). Performances were assessed in terms of xylose uptake rate (blue bars representing ratios of isolate to parent) and ethanol yield per sugar supplied (green bars representing ratios of isolate to parent). Isolates from ethanol-challenged continuous cultures on xylose were tested in this screen using PSGHL supplemented with glucose (C and D) in order to allow formation of significant ethanol to more strongly challenge induction of xylose utilization enzymes and metabolism of xylose.

Isolates from all phases of the adaptation process were screened in PSGHL as the primary elimination point for those cultures not able to ferment xylose well. The isolates chosen for representation in Figure 8 are among the best performing on PSGHL out of the approximately 150 ranked in this primary screen and in all secondary phases of performance ranking described below. In order to indicate improvement relative to the parent strain, the performance of each isolate was expressed as the ratio of isolate kinetic parameter value to parent strain NRRL Y-7124 kinetic parameter value. Ratio values of ‘one’ occurred if the isolate performance, based on either yield or xylose uptake rate, was equivalent to the parent. Ethanol yield per initial sugar available and xylose uptake rate ratios were used to rank relative performances of isolates.

Some general trends in isolate responses to harshness of the primary hydrolyzate screen were notable. As the harshness of the hydrolyzate environment was increased with inhibitor and glucose concentrations, the performance ratios became progressively smaller as shown in Figure 8a-d, respectively. Figure 8a,b provides a summary of top isolate performances on 60% and 75% strengths of PSGHL with ODM and ODM + YM nutrient supplements, respectively. In the 60% strength PSGHL, five of seven top isolates exposed to PSGHL selection pressure performed many times better than NRRL Y-7124 (isolate 1), and four isolates performed slightly better than colony 5 (isolate 33), which had evolved during exposure to AFEX CSH but had no previous selective exposure to PSGHL. However, in the harsher 75% strength of PSGHL, only three isolates significantly surpassed both the parent and colony 5 (isolate 33) despite improved nutrient richness.

In a related experiment, isolates obtained from the continuous culture challenged with xylose growth in the presence of ethanol (isolates 27, 28, and 30) were screened on similar PSGHL media that were also supplemented with 75 g/L glucose (Figure 8c,d). The glucose addition was chosen to heighten the ethanol repression challenge to xylose utilization in order to detect isolates with the conserved reduced diauxy feature characteristic of the parent colony 5 (isolate 33) from which they arose, as well as general ethanol resistance to cell damage. However, these isolates had not previously been exposed to PSGHL, and, as shown in Figure 8c,d, they struggled to surpass par with the NRRL Y-7124 parent even when the nutrient environment was enriched with YM components. Thus, Figure 8 indicates that the relative ranking of isolates on a particular culture medium, such as PSGHL, could vary due to the prior adaptation medium exposure, the strength of the hydrolyzate, and the nutrients applied.

### Impact of nutrient environment on differentiation of isolates in a secondary screen on enzyme-saccharified hydrolyzates

When isolates passing the primary screen were submitted to a secondary screen on unamended SGH without any nitrogen supplementation, all isolates performed poorly, and there was no significant variation (*P* > 0.05). Across the 33 isolates tested, including the parent NRRL Y-7124, the kinetic parameter means and standard deviations were 0.19 ± 0.1 g/g for ethanol yield per initial sugar and 0.10 ± 0.03 g/L/h, for xylose uptake rate.

Past studies on defined media have shown that optimal xylose fermentation by *S. stipitis* strain NRRL Y-7124 requires C:N 57 to 126:1, and 20% to 60% of available nitrogen must be in the form of amino acids while 80% to 40% must be urea [10]. Higher amino acid contents may be required in the presence of hydrolyzate inhibitors, especially during xylose fermentation [10,24]. The C:N ratio of the typical SGH used in this study was approximately 600:1, and nitrogen starvation would result if not supplemented. Since nitrogen availability and quality have been shown to impact the growth and fermentation capacity of the parent NRRL Y-7124, SGH was supplemented with both urea and amino acids. SGH-N1 was fortified to 42:1 C:N (approximately 15% of N from PAN and approximately 85% as urea) with complex nitrogen sources and growth factors including casamino acids, tryptophan, cysteine, and vitamins. The SGH-N2 was fortified to 37:1 C:N with urea and soy flour as the lowest cost commercial source of PAN and vitamins, providing approximately 12% of N from PAN and 88% as urea. Thus, the two SGH nitrogen environments -N1 and -N2 were close, but below the ideal. The 6% glucan AFEX CSH had the optimal mix of ammonia and amino acids but a C:N at approximately half of the optimal 60:1 feed in defined media.

**Figure 9:**
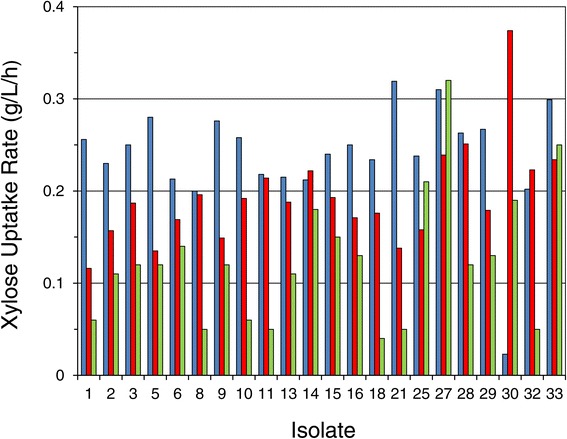
Significant dependence of xylose uptake rate on the interaction of isolate with hydrolyzate type (*P* < 0.001). Isolates (identified along x-axis) were tested in a secondary screen on two nutrient formulations of switchgrass hydrolyzate, SGH-N1 (blue bars) and SGH-N2 (red bars), and additionally AFEX-pretreated corn stover hydrolyzate AFEX CSH (green bars) without nutrient supplementation.

While overall C:N and PAN:ammonia content are easily measurable, the hydrolyzate environments are complex and likely to vary in amino acid, growth factor, and mineral profiles, which may potentially impact fermentations. The strategy for the secondary isolate screen then was to apply all three nutritional environments in search of isolates with the nutritional flexibility to perform well and consistently despite the potential for nutritional variability to occur under commercial circumstances. A two-way ANOVA testing the impact of isolates × hydrolyzate types on yield (data not shown) and xylose uptake rate (Figure 9) was carried out on data collected from hydrolyzate screen conducted in duplicate. When superior isolates passing the primary screen on PSGHL were evaluated on the three hydrolyzate types (SGH-N1, SGH-N2, and AFEX CSH), the overall mean xylose uptake rate across isolates varied significantly between hydrolyzate types (*P* < 0.001): 0.24 g/L/h for SGH-N1, 0.19 g/L/h for SGH-N2, and 0.12 g/L/h for AFEX CSH. This interaction is evident in the data presented in Figure 9. The relative xylose consumption rates among isolates were significantly dependent on the hydrolyzate type used in the screen, such that there was a nearly significant dependence on isolate (*P* = 0.058) and a strongly significant interaction of isolate × hydrolyzate type impacting xylose uptake rate (*P* < 0.001). In contrast, the ethanol yield (data not shown) was not significantly impacted by hydrolyzate type (*P* = 0.967) or isolate (*P* = 1). The overall yield mean and standard deviation were 0.30 ± 0.03 g ethanol/g initial ethanol per g initial sugar supplied.

### Ranking of single-cell isolates from AFEX CSH, ethanol, and PSGHL adaptation phases

Relative performance indices (RPIs) were calculated in order to rank isolates in sets within a series of different experiments based on their relative performance in a variety of hydrolyzates and nutritional environments tested. RPI is a dimensionless value that is useful in combining data sets to use in overall ranking and/or statistical analysis of subjects submitted to various testing procedures. In this case, yeast isolates were ranked based on ethanol yield per initial sugar available and xylose uptake rate within various experiment sets. Given that the parameters calculated for the performance of each isolate on each hydrolyzate were normally distributed across the group of isolates tested, the value of *F* = (*X* − *X*_avg_)/*s* ranges from −2 to +2. Here, *X* can designate yield (*Y*) or rate (*R*) observed per isolate, and *X*_avg_ and *s* are the average and standard deviation, respectively, of all values observed for the group of isolate treatments within a given experiment, such as the testing of isolates on AFEX CSH. RPI = (2 + *F*) × 100/4, such that the value of RPI ranges from approximately 0 to 100 percentile from lowest to highest value, respectively, of rate or yield. RPI averages for each isolate within a given hydrolyzate type trial were calculated as RPI = (RPI_Y_ + RPI_R_)/2, where yield and rate contributions (subscripted *Y* and *R*, respectively) are given equal weighting in this application. (In general, pending the application and relative importance, different weights could be rationally assigned to the yield and rate contributions to the overall RPI average.) Additionally in this study, RPI_overall_ was computed across the *n* types of hydrolyzates tested: RPI_overall_ = [(RPI_Y_ + RPI_R_)_1_ + (RPI_Y_ + RPI_R_)_2_ +  . . . (RPI_Y_ + RPI_R_)_*n*_ ]/2*n*. During primary ranking of the approximately 150 single-colony isolates adapted to xylose-rich PSGHL, the RPI_overall_ was first calculated for rates and yields across the two PSGHL formulations applied in the screen, that is, 60% PSGHL and 75% PSGHL. During secondary screening of the top 20% of isolates from the primary screen, the RPI_overall_ was next similarly calculated for each of the isolates performing on three enzyme-saccharified pretreated hydrolyzate formulations including AFEX CSH, SGH-N1, and SGH-N2, and this ranking parameter was applied to further winnow the list of superior isolates.

**Figure 10:**
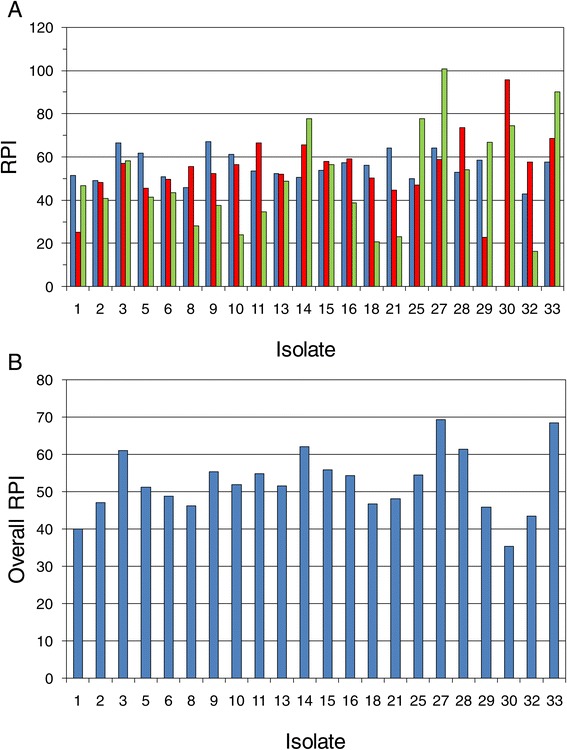
Isolate ranking based on RPI. The relative performance index (RPI) concept was applied to the performance results of the secondary screen in order to rank 33 isolates within each hydrolyzate type based on xylose uptake rate and ethanol yield per sugar supplied. (**A**) The relative ranking of any given isolate depended on the hydrolyzate type (*P* < 0.001): SGH-N1 (blue bars), SGH-N2 (red bars), and AFEX CSH (green bars). (**B**) The overall RPI calculated across all hydrolyzate types (light blue bars) indicated superior strains with most robust performance across different hydrolyzate conditions.

**Figure 11:**
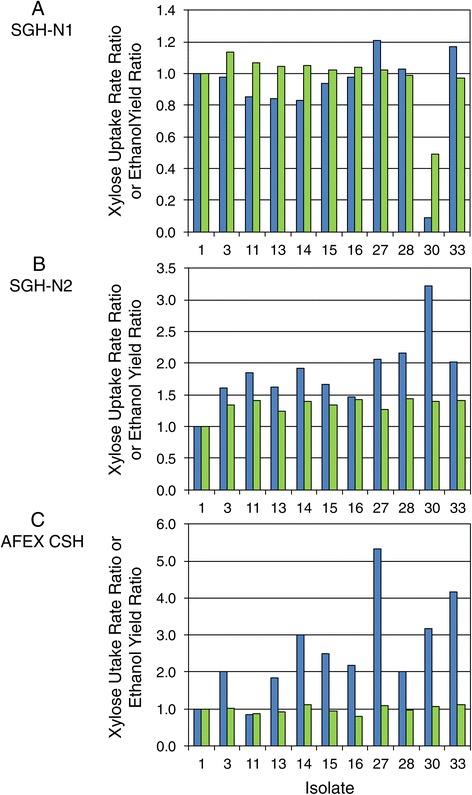
Performance summary of superior adapted isolates of *S. stipitis.* Isolates were screened on three hydrolyzate types, including switchgrass hydrolyzate with two nutrient formulations and unsupplemented AFEX-pretreated corn stover hydrolyzate: (**A**) SGH-N1, (**B**) SGH-N2, and (**C**) AFEX CSH. The improvement in superior adapted isolates over the parent strain was hydrolyzate dependent and is indicated in terms of the xylose uptake rate ratio (dark blue bars) or the ethanol yield ratio (light green bars), which are ratios of adapted strain to parent strain NRRL Y-7124 kinetic parameter values.

Figure 10a shows RPI results within the three different hydrolyzate types for a selection of the better isolates among the 33 tested in the secondary screen. A two-way ANOVA indicated significant variation in RPI due to isolate (*P* = 0.003); but since RPI values were scaled within each hydrolyzate type relative to the data set mean and standard deviation, the mean RPI did not significantly vary among the three hydrolyzates tested (*P* = 0.27), which had mean RPIs ranging from 50 to 55. However, the interaction of isolate × hydrolyzate type was strongly significant (*P* < 0.001), and the relative ranking of isolates depended upon the type of hydrolyzate. This variation in ranking may have come about because of variations in isolate nutritional requirements or inhibitor sensitivities, but it also may have arisen due to instability of certain isolates and inconsistencies even within the same type of hydrolyzate. The identification of isolates with highest overall RPI and lowest relative standard deviation among rankings on different hydrolyzates and replicates was a goal of our analysis. Such isolates possess broad inhibitor tolerance, nutritional diversity, and genetic stability - all characteristics useful toward commercial robustness. Five such superior isolates with RPI >60 are indicated in Figure 10b: 3, 14, 27, 28, and 33. Another strategy was to identify any isolates that were specialists or that consistently ranked highly RPI >55 within a hydrolyzate type or overall with a low relative standard deviation: 11 (SGH), 13, 15, 16 (SGH), and 30 (SGH-N2) as indicated in Table 3 and Figure 10. Most superior isolates fell within statistics group A or B (as shown in Table 3), but isolate 30 was in group D since it was very good in SGH-N2 and AFEX CSH but very poor in SGH-N1. Figure 11 indicates the improvement of each of the superior isolates over the unadapted parent NRRL Y-7124 and shows that isolate abilities were best separated by fermentation on AFEX CSH and SGH-N2. AFEX CSH supported the highest dynamic range of improvement in xylose uptake rate among isolates tested, but SGH-N2 supported the highest range of improvement in the ethanol yield per initial sugar supplied. Isolate abilities were not very distinguishable from one another on SGH-N1 (the least cost effective) perhaps because it was the least challenging since it was the most nutritionally fortified with added yeast extract, malt extract, amino acids, vitamins, and minerals.

**Table 3:** **Summary of relative performance indexes (RPI**_**overall**_ **) of isolates**

		**SGH-N1 and SGH-N2**				**SGH-N1, SGH-N2, and AFEX CSH**			
**Screen number**	**Isolate designation**	**RPI**_**overall**_	**s**	**Rel. s (%)**	**Statistic group**	**RPI**_**overall**_	**s**	**Rel. s (%)**	**Statistic group**
1	Y-7124	38.3	23.6	61.5	D	40.0	23.0	57.3	C
2	Y-7124 S80E40-2	48.6	24.7	50.8	C	47.0	22.2	47.1	C
*3*	*Y-7124 S90E40-1*	*61.8*	*15.6*	*25.2*	*A*	*61.1*	*14.7*	*24.0*	*A*
4	Colony 5 S90E40-5	40.1	12.6	31.5	C				
5	Colony 5 S100E40-5	53.6	11.8	22.0	B	51.1	12.3	24.0	B
6	2A.1.53R S90E40-4	54.0	19.0	35.2	B	48.8	12.4	25.5	C
7	2A.1.53R S100E40-5	52.3	16.6	31.7	B				
8	Y-7124 GP-1	50.7	23.9	47.1	B	46.2	23.1	50.1	C
*9*	*Y-7124 GP-5*	*59.8*	*15.7*	*26.3*	*B*	*55.3*	*17.6*	*31.9*	*B*
10	Colony 5 GP-2	58.9	10.1	17.2	B	51.9	17.4	33.5	B
*11*	*Colony 5 GP-6*	*59.9*	*15.4*	*25.8*	*B*	*54.8*	*17.7*	*32.3*	*B*
12	2A.1.53R S90E40-2	34.2	17.1	50.1	D				
*13*	*2A.1.53R S100E40-1*	*52.2*	*10.5*	*20.1*	*B*	*51.5*	*9.5*	*18.4*	*B*
*14*	*Y-7124-6*	*58.2*	*16.2*	*27.9*	*B*	*62.1*	*17.0*	*27.3*	*A*
*15*	*Y-7124-10*	*57.6*	*6.6*	*11.4*	*B*	*55.9*	*6.7*	*11.9*	*B*
*16*	*2A.1.53R-1*	*58.3*	*10.2*	*17.4*	*B*	*54.4*	*14.0*	*25.8*	*B*
17	2A.1.53R-6	49.4	13.1	26.5	C				
18	Colony 5-3	53.3	11.7	22.0	B	46.7	17.2	36.8	C
19	Colony 5-4	41.6	16.7	40.1	C				
20	Y-7124 GP3-1	32.0	18.2	56.8	D				
21	Y-7124 GP3-5	54.4	18.9	34.7	B	48.1	21.2	44.1	C
22	Colony 5 25%-2 N	46.8	29.3	62.6	C				
23	2A.1.53R 25%-1 N	47.8	13.8	28.9	C				
24	2A.1.53R 25%-2	51.5	16.7	32.4	B				
25	2A.44R-E20-C1	48.6	15.4	31.7	C	54.4	18.4	33.8	B
26	2A.44R-E40-C2	55.0	29.7	54.0	B				
*27*	*2A.1.53R-E20-C1*	*61.5*	*23.7*	*38.6*	*A*	*69.3*	*27.7*	*39.9*	*A*
*28*	*2A.1.53R-E30-C3*	*63.3*	*17.6*	*27.9*	*A*	*61.4*	*16.2*	*26.4*	*A*
29	2A.30R2-E30-C5	40.5	52.9	130.6	C	45.8	48.5	105.9	C
*30*	*2A.30R2-E40-C5*	*25.5*	*81.4*	*319.3*	*D*	*32.3*	*74.7*	*231.4*	*D*
31	3A.1.57-E20-C1	32.2	13.7	42.5	D				
32	3A.1.57-E30-C1	50.2	11.2	22.3	B	43.4	17.7	40.8	C
*33*	*Colony 5*	*63.0*	*14.8*	*23.5*	*A*	*68.4*	*17.4*	*25.4*	*A*

Isolates in italics are considered superior based on high overall RPI across hydrolyzate types, low relative standard deviation (Rel. s), and/or exceedingly high RPI on at least one hydrolyzate type as seen in Figure 10A.

Strains are ranked based on ethanol yield and xylose uptake rate in screens on dilute acid-pretreated switchgrass enzyme hydrolyzates with two different nutrient supplements (SGH-N1 and SGH-N2) or over all three hydrolyzates including AFEX-pretreated corn stover enzyme hydrolyzates.

### Comparative kinetic performances of isolates recovered after varied adaptation stress

#### Reduction of diauxic lag

Significant ethanol production can occur on AFEX CSH prior to xylose uptake due to the presence of approximately 60 g/L glucose in this hydrolyzate, and this presents a challenge to induction of enzymes for xylose utilization. After exposure to AFEX CSH selection pressure, colony 5 displayed significantly reduced diauxic lag (Figure 3). Consequently, the induction of xylose utilization in cultures inoculated to low initial cell densities (A_620.0_ of 0.1) on ODM with 75 g/L each of xylose and glucose were evaluated on a selection of the superior strains in order to check the occurrence of the reduced diauxy phenotype in PSGHL-adapted isolates and its retention in derivatives of colony 5 (AFEX CSH) obtained after exposure to ethanol-challenged continuous cultures and/or PSGHL. The data summarized in Table 4 indicated that while glucose uptake rate remained the same among all isolates, the reduced diauxy trait was evidenced by faster xylose uptake rates (and higher ethanol productivities on xylose) in colony 5 recovered from AFEX CSH adaptation, both AFEX CSH > E isolates with or without UV exposure and two of three PSGHL-evolved strains. However, the trait was notably lost from all AFEX CSH treatments later exposed to repetitive culturing in PSGHL. Unlike AFEX CSH, PSGHL is rich in xylose and high in furfural and acetate but poor in glucose, and so during exposure, there would be little selective pressure favoring reduced diauxy due to <5 g/L ethanol formed prior to xylose uptake. This situation may have led to loss of the trait after extended repetitive culturing of AFEX CSH +/− E derivatives in PSGHL. However, two of three PSGHL stressed isolates gained a similar reduced diauxy phenotype. Repeated exposure to xylose among the other inhibitors present, such as acetic acid and furan aldehydes, may have benefited the evolution process toward reduced diauxy but likely by different mechanisms than during exposure to AFEX CSH. For example, during PSGHL exposure, furfural and HMF could potentially compete with xylose for reducing equivalents [25-27] while acetate would cause cell damage, forcing the need for cells to metabolize xylose to accommodate repair of cell damage.

**Table 4:** **Comparative performance of superior isolates on ODM + 74.5 g/L xylose + 77.6 g/L glucose inoculated to low cell density (A**_**620**_ **= 0.1)** ^**a**^

			**Sugar uptake rate (g/L/h)**				**Ethanol productivity (g/L/h)**				**Yield per initial sugar (g/g)**					
**Adaptation stress**	**Isolate**		**Glucose**		**Xylose**		**Glucose**		**Xylose**		**Ethanol**		**Biomass**		**Xylitol**	
AFEX CSH	33	Colony 5	1.08	A	0.38	A	0.48	A	0.14	AB	0.37	A	0.016	BC	0.018	B
AFEX CSH > E	28	2A.1.53R-E30-C3	1.09	A	0.38	A	0.48	A	0.15	A	0.37	A	0.020	AB	0.018	B
AFEX CSH > E (UV)	30	2A.1.30R2-E40-C5	1.10	A	0.38	A	0.48	A	0.13	AB	0.35	AB	0.022	A	0.029	A
AFEX CSH > E > PSGHL	13	2A.1.53R-S100E40-1	0.96	A	0.30	C	0.41	AB	0.12	ABC	0.32	BC	0.014	BC	0.011	C
AFEX CSH > E > PSGHL	16	2A.1.53R-1	1.05	A	0.27	C	0.41	AB	0.09	C	0.29	C	0.012	CD	0.015	BC
AFEX CSH > PSGHL	11	Colony 5-GP6	0.91	A	0.27	C	0.37	B	0.11	BC	0.30	C	0.010	D	0.010	C
PSGHL	3	Y-7124-S90E40-1	1.00	A	0.38	A	0.41	AB	0.15	A	0.35	AB	0.013	CD	0.026	A
PSGHL	14	Y-7124-6	0.97	A	0.34	B	0.38	B	0.13	AB	0.32	BC	0.017	BC	0.018	B
PSGHL	15	Y-7124-10	1.08	A	0.29	C	0.44	AB	0.11	BC	0.30	C	0.009	D	0.012	C
Wild	1	Y-7124	1.05	A	0.29	C	0.48	A	0.11	BC	0.34	ABC	0.019	AB	0.010	C

^a^With the exception of glucose uptake rate, all parameters varied significantly among isolates based on one-way ANOVA (*P* < 0.01). Within columns, values with no letters in common are significantly different at *P* < 0.05 (Student-Newman-Keuls pairwise comparison method).

#### Acetic acid tolerance

Parent strain NRRL Y-7124 and hydrolyzate-tolerant derivatives showed little difference in their abilities to grow on xylose or glucose in the presence of acetic acid at 6 to 15 g/L (Table 5). Specific growth rates in the absence of acetic acid were similar, ranging from 0.23 to .28 h^−1^ on glucose and from 0.22 to .26 h^−1^ on xylose. However, the results of a three-way ANOVA conducted on the ratio of specific growth rate in the presence of inhibitory acetic acid to that in the absence of acetic acid (μ_*i*_/μ_*o*_) showed that cells growing on xylose were significantly more inhibited by acetic acid (growth rates reduced to 68% by 15 g/L acetic acid) than cells growing on glucose (growth rates reduced to just 79%). Isolate 30 (2A.1.30R2-E40-C5) was significantly more resistant to acetic acid on xylose (average μ_*i*_/μ_*o*_ = 1.1) than all other strains and among the most resistant to acetic acid on glucose (average μ_*i*_/μ_*o*_ = 0.93).

**Table 5:** **Comparative average resistances of superior isolates to 6, 10, and 15 g/L acetic acid in ODM with 50 g/L sugar** ^**a,b**^

**Adaptation stress**	**Isolate**		**Average μ**_***o***_ **(h** ^**−1**^ **)** ^**c**^				**Average ratio μ**_***i***_ **/μ**_***o***_^**c**^			
			**Glucose**		**Xylose**		**Glucose**		**Xylose**	
AFEX CSH	33	Colony 5	0.23	AB	0.23	A	0.86	AB	0.90	B
AFEX CSH > E	27	2A.1.53R-E20-C1	0.28	A	0.26	A	0.72	AB	0.80	B
AFEX CSH > E	28	2A.1.53R-E30-C3	0.26	AB	0.24	A	0.75	AB	0.68	B
AFEX CSH > E (UV)	30	2A.1.30R2-E40-C5	0.24	AB	0.21	A	0.93	AB	1.10	A
AFEX CSH > E > PSGHL	13	2A.1.53R S100E40-1	0.26	AB	0.25	A	0.84	AB	0.79	B
AFEX CSH > E > PSGHL	16	2A.1.53R-1	0.25	AB	0.24	A	0.88	AB	0.75	B
AFEX CSH > PSGHL	11	Colony 5-GP6	0.22	B	0.25	A	1.02	A	0.84	B
PSGHL	3	Y-7124 S90E40-1	0.25	AB	0.22	A	0.66	B	0.83	B
PSGHL	15	Y-7124-10	0.25	AB	0.24	A	0.97	A	0.85	B
PSGHL	14	Y-7124-6	0.23	AB	0.23	A	0.84	AB	0.81	B
Wild	1	Y-7124	0.24	AB	0.25	A	0.96	AB	0.87	B
Significance			*P* = 0.039		*P* = 0.223		*P* < 0.01		*P* < 0.001	
**Acetic acid (g/L)**	**Average ratio μ**_***i***_ **/μ**_***o***_^**d**^									
	**Glucose**		**Xylose**							
6	0.98	A	0.98	A						
10	0.80	B	0.89	B						
15	0.79	B	0.64	C						
Significance	*P* < 0.001		*P* < 0.001							

^a^Abbreviations are the following: μ_*o*_ = initial specific growth rate in the absence of acetic acid; μ_*i*_ = the specific growth rate in the presence of the inhibitory acetic acid.

^b^Within columns, values with no letters in common are significantly different at *P* < 0.05 (Student-Newman-Keuls pairwise comparison method).

^c^Average across acetic acid concentrations for a particular isolate.

^d^Average across isolates for a particular acetic acid concentration.

Fermentation of 75 g/L xylose in ODM by large populations was significantly more inhibited as acetic acid was increased from 5 to 15 g/L. The level of inhibition was significantly higher when the cell populations were glucose grown rather than xylose grown, suggesting the increased difficulty of glucose-using cells to switch to xylose utilization when under stress by acetic acid (Table 6). The overall impact of acetic acid across isolates was reflected by significant differences in xylose uptake rate, ethanol productivity, and ethanol yield. When strain populations were pre-grown on xylose, the xylose uptake and ethanol production rates and yields were higher than the parent NRRL Y-7124 for all strains that had been developed with AFEX CSH stress. The three strains that had received only PSGHL adaptation stress fermented xylose more slowly than the parent strain NRRL Y-7124 (Table 6A). On the contrary, when the cell populations were grown on glucose, all strains that had been exposed to only PSGHL adaptation stress fermented xylose significantly faster and at significantly higher ethanol yield than when they were grown on xylose (Table 6B).

**Table 6:** **Impact of acetic acid on xylose fermentation by large isolate populations (A**_**620**_ **= 50)**

**A. Comparative fermentation of 75 g/L xylose in ODM with 5 to 15 g/L acetic acid by isolates precultured on xylose**								
**Adaptation stress**	**Isolate**		**Xylose uptake rate (g/L/hA)** ^**a,b**^		**Ethanol productivity (g/L/hA)** ^**a,b**^		**Ethanol yield per initial sugar (g/g)** ^**b**^	
AFEX CSH	33	Colony 5	0.090	A	0.0231	AB	0.26	B
AFEX CSH > E	28	2A.1.53R-E30-C3	0.078	AB	0.0197	ABC	0.23	BCD
AFEX CSH > E (UV)	30	2A.1.30R2-E40-C5	0.071	BC	0.0155	ABCDE	0.20	DE
AFEX CSH > E > PSGHL	13	2A.1.53R S100E40-1	0.069	BC	0.0249	A	0.23	BCD
AFEX CSH > E > PSGHL	16	2A.1.53R-1	0.078	AB	0.0175	ABCD	0.25	BC
AFEX CSH > PSGHL	11	Colony 5-GP6	0.066	BC	0.0141	BCDE	0.21	CD
PSGHL	3	Y-7124 S90E40-1	0.043	D	0.0080	DE	0.14	F
PSGHL	14	Y-7124-6	0.053	CD	0.0105	CDE	0.16	EF
PSGHL	15	Y-7124-10	0.045	D	0.0061	E	0.30	A
Wild	1	Y-7124	0.060	C	0.0148	BCDE	0.19	DE
	**Acetic acid (g/L)**							
	5		0.083	A	0.0223	A	0.29	A
	10		0.065	B	0.0152	B	0.22	B
	15		0.048	C	0.0088	C	0.14	C
**B. Comparative fermentation of 75 g/L xylose in ODM with 5 to 15 g/L acetic acid by isolates precultured on glucose**								
**Adaptation stress**	**Isolate**		**Xylose uptake rate (g/L/hA)** ^**a,b**^		**Ethanol productivity (g/L/hA)** ^**a,b**^		**Ethanol yield per initial sugar (g/g)** ^**b**^	
AFEX CSH	33	Colony 5	0.014	C	0.0027	B	0.09	C
AFEX CSH > E	28	2A.1.53R-E30-C3	0.018	C	0.0039	B	0.09	C
AFEX CSH > E (UV)	30	2A.1.30R2-E40-C5	0.029	BC	0.0094	AB	0.16	BC
AFEX CSH > E > PSGHL	13	2A.1.53R S100E40-1	0.021	C	0.0047	B	0.18	B
AFEX CSH > E > PSGHL	16	2A.1.53R-1	0.019	C	0.0044	B	0.16	BC
AFEX CSH > PSGHL	11	Colony 5-GP6	0.014	C	0.0049	B	0.15	BC
PSGHL	3	Y-7124 S90E40-1	0.058	A	0.0165	A	0.26	A
PSGHL	14	Y-7124-6	0.044	AB	0.0108	AB	0.27	A
PSGHL	15	Y-7124-10	0.050	A	0.0141	A	0.23	A
Wild	1	Y-7124	0.018	C	0.0050	B	0.13	BC
	**Acetic acid (g/L)**							
	5		0.043	A	0.0126	A	0.24	A
	10		0.028	B	0.0071	B	0.17	B
	15		0.014	C	0.0032	C	0.10	C

^a^Rates are normalized relative to population density in absorbance units (A) at 620 nm.

^b^Parameter variations based on two-way ANOVA (isolate × acetic ) were significant (*P* < 0.001). Within columns, values with no letters in common are significantly different at *P* < 0.05 (Student-Newman-Keuls pairwise method).

#### Furfural tolerance

**Figure 12:**
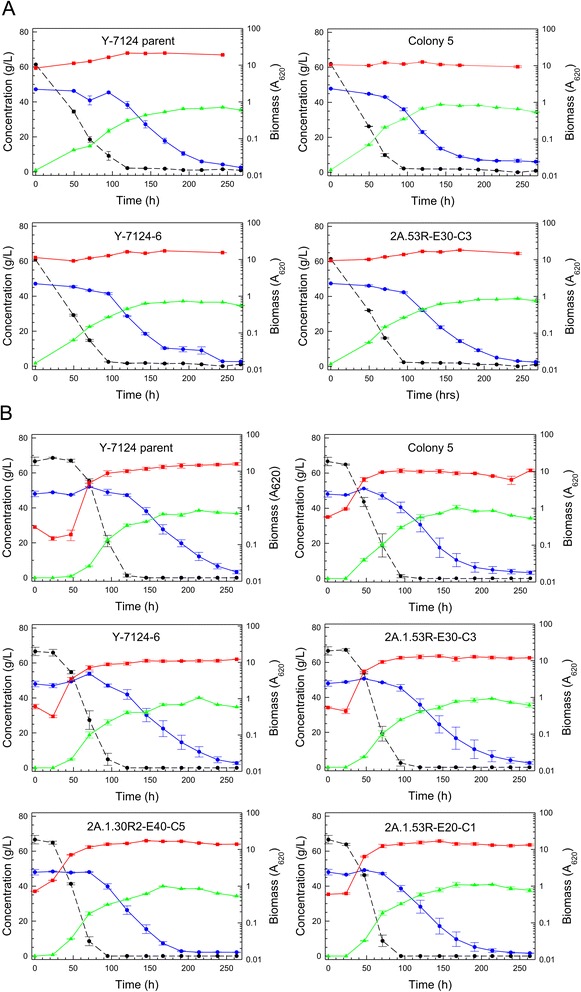
Comparative SGH fermentations of superior adapted isolates of *S. stipitis*. Superior adapted isolates and their parent strain NRRL Y-7124 are compared fermenting enzymatic hydrolyzates of dilute acid-pretreated switchgrass (20% solids loading) at 25°C and initial pH 6.2 at high initial cell density (**A**) or low initial cell density (**B**). Time courses of biomass (red squares), glucose (black circles and dashed line), xylose (blue circles and solid line), and ethanol (green triangles) are shown. Error bars represent the range about the mean value marked by symbols.

The furfural content of the PSGHL used in adaptation cultures was around 24 mM with little accompanying HMF (Table 1). Furfural is typically reduced enzymatically with NADH cofactors by yeasts to less toxic furan methanol [25-27]. When the parent NRRL Y-7124 and hydrolyzate-tolerant derivatives were challenged to grow in ODM + 50 g/L glucose (or xylose) amended with 25 mM distilled furfural, all were able to survive and begin growing within 32.8 to 42.1 h (data not shown). Past reports have suggested that xylose and low level furan utilization may be compatible due to the competition for NADH limiting xylitol accumulation [28]. Statistical analysis of our results indicated some benefit of xylose shortening lag phase as a general trend across all isolates, but only by approximately 2.5 h. Considering glucose as the growth substrate most likely to be encountered during detoxification lag, one exceptional isolate, 2A.1.30R2-E40-C5 (AFEX CSH > E (UV)) had a significantly shorter lag phase of 32.7 h compared with that of the parent NRRL Y-7124 at 37.6 h. It is also notable that the growth of isolate 2A.1.30R2-E40-C5 was not reduced in the presence of up to 15 g/L acetic acid (Table 5). These attributes may give this strain a competitive advantage in hydrolyzate fermentations as seen later in Figure 12B. Interestingly, strain 2A.1.30R2-E40-C5 was exposed to AFEX CSH, high levels of ethanol with xylose in extended continuous cultures, and UV irradiation during its development, but its exposures to furfural and acetate were only low to moderate during the hydrolyzate phase, suggesting that adaptive changes were generally useful to coping with stress from inhibitors. Additionally, the finding that most adapted isolates were in general not especially faster at detoxifying furfural in defined medium compared with parent NRRL Y-7124 suggests that other attributes and mechanisms were involved in aiding their ability to cope in hydrolyzates.

#### Hydrolyzate utilization

When moderately high cell densities were applied initially to inoculate larger flask cultures with SGH-N2, almost all of the adapted strains ranked as superior in our screen were consuming sugars more quickly and producing ethanol more quickly on a volumetric rate basis (Table 7A) compared to the control, wild type strain Y-7124. To reduce the influence of cell biomass variations due to growth advantages of some strains, especially during glucose consumption, rates were normalized based on the average absorbance reading during glucose or xylose uptake (Table 7B). This procedure further enhanced statistical separation among strains, especially with respect to xylose uptake and ethanol productivity on xylose. This procedure indicated NRRL Y-7124 S90E40-1 as having particularly high fermentation capacity on a specific rate basis although its ability to grow and accumulate biomass in the full strength hydrolyzate was weaker than for other strains. The ethanol yields of 0.31 to 0.34 g/g initial sugar did not vary significantly among strains (Table 7C). In Figure 12A, corresponding to the kinetic data of Table 7, time courses are shown for the parent NRRL Y-7124 and selected adapted strains representing the most successful under these conditions: colony 5 (AFEX CSH), Y-7124-6 (PSGHL), and 2A.53R-E30-C3 (AFEX CSH > E). Highest ethanol accumulations reached 39 g/L for adapted strains compared to 36 g/L for the parent strain.

**Table 7:** **Comparative kinetics of isolates on switchgrass hydrolyzate SGH-N2 inoculated to A**_**620**_ **= 8.4 +/− 2.5** ^**a**^

**A. Volumetric rates during glucose or xylose consumption**												
**Adaptation stress**	**Isolate**		**Sugar uptake rate (g/L/h)**				**Ethanol productivity (g/L/h)**					
			**Glucose**		**Xylose**		**Glucose**		**Xylose**		**Overall**	
AFEX CSH	33	Colony 5	0.65	A	0.19	D	0.32	A	0.174	A	0.26	A
AFEX CSH > E	28	2A.1.53R-E30-C3	0.63	A	0.27	B	0.29	A	0.076	C	0.16	D
AFEX CSH > E > PSGHL	13	2A.1.53R S100E40-1	0.43	C	0.27	B	0.20	C	0.089	C	0.15	E
AFEX CSH > E > PSGHL	16	2A.1.53R-1	0.57	AB	0.23	C	0.27	AB	0.083	C	0.17	D
PSGHL	3	Y-7124 S90E40-1	0.65	A	0.16	E	0.30	A	0.140	B	0.24	B
PSGHL	14	Y-7124-6	0.62	A	0.24	C	0.28	AB	0.090	C	0.18	C
PSGHL	15	Y-7124-10	0.63	A	0.20	D	0.30	A	0.066	C	0.16	D
Wild	1	Y-7124	0.51	B	0.30	A	0.24	B	0.059	C	0.15	E
**B. Normalized rates per unit absorbance (620 nm) during glucose or xylose consumption**												
**Adaptation stress**	**Isolate**		**Sugar uptake rate (g/L/hA)**				**Ethanol productivity (g/L/hA)**					
			**Glucose**		**Xylose**		**Glucose**		**Xylose**		**Overall**	
AFEX CSH	33	Colony 5	0.060	D	0.017	B	0.029	C	0.0161	B	0.024	B
AFEX CSH > E	28	2A.1.53R-E30-C3	0.056	D	0.017	B	0.025	CD	0.0048	DF	0.012	C
AFEX CSH > E > PSGHL	13	2A.1.53R S100E40-1	0.040	E	0.019	B	0.018	D	0.0063	CDF	0.012	C
AFEX CSH > E > PSGHL	16	2A.1.53R-1	0.082	C	0.026	A	0.039	B	0.0094	C	0.022	B
PSGHL	3	Y-7124 S90E40-1	0.152	A	0.025	A	0.072	A	0.0216	A	0.043	A
PSGHL	14	Y-7124-6	0.057	D	0.016	B	0.026	C	0.0059	CDF	0.014	C
PSGHL	15	Y-7124-10	0.089	B	0.023	A	0.042	B	0.0076	CD	0.020	B
Wild	1	Y-7124	0.037	E	0.014	B	0.017	D	0.0028	F	0.009	D
**C. Yields and residuals per total initial sugar**												
**Adaptation stress**	**Isolate**		**Yields or residuals per total initial sugar (g/g)**									
			**Ethanol**		**Biomass**		**Xylitol**		**Residual xylose**			
AFEX CSH	33	Colony 5	0.34	A	0.0030	D	0.025	A	0.055	B		
AFEX CSH > E	28	2A.1.53R-E30-C3	0.34	A	0.0134	B	0.020	A	0.023	C		
AFEX CSH > E > PSGHL	13	2A.1.53R S100E40-1	0.31	B	0.0067	D	0.024	A	0.060	B		
AFEX CSH > E > PSGHL	16	2A.1.53R-1	0.34	A	0.0045	D	0.019	A	0.096	A		
PSGHL	3	Y-7124 S90E40-1	0.32	B	0.0060	D	0.023	A	0.113	A		
PSGHL	14	Y-7124-6	0.33	AB	0.0091	C	0.021	A	0.024	C		
PSGHL	15	Y-7124-10	0.33	AB	0.0050	D	0.029	A	0.073	B		
Wild	1	Y-7124	0.33	AB	0.0165	A	0.026	A	0.023	C		

^a^With the exception of xylitol yield, all parameters varied significantly among isolates based on one-way ANOVA (*P* < 0.001). Within columns, values with no letters in common are significantly different at *P* < 0.05 (Student-Newman-Keuls pairwise comparison method).

A similar flask experiment was run to compare kinetics in cultures inoculated to a low initial cell density (Figure 12B). In this situation, the ability of strains to grow in the hydrolyzate and transition to fermentation was tested. Time courses of the control and selected adapted strains are shown in Figure 12B, and here the parent strain performed particularly poorly relative to all of the adapted strains because it suffered a 48-h lag period before growth began whereas the lag for adapted strains was much shorter at 24 h or less. Highest ethanol accumulations reached were 42 g/L at 167 h for adapted strains compared to 38 g/L at 213 h for the parent NRRL Y-7124. For the adapted strains, 85% of maximum ethanol accumulation was reached by 120 h. These kinetics are the most favorable ever reported for high solids loading hydrolyzates inoculated with non-engineered yeast strains at pH 5 to 6 without prior detoxification.

## Conclusions

The application of single-phase and multi-phase adaptation schemes led to the recovery of significantly improved strains of *S. stipitis* with genetic stability indicated by performance consistency in defined and hydrolyzate-challenged culture media. The AFEX CSH adaptation was sufficient to cause significant improvement of the performance of *S. stipitis* in enzyme hydrolyzates of both AFEX-pretreated corn stover and dilute acid-pretreated switchgrass. This improvement, especially with respect to reduced diauxic lag during sugar transition, was further stabilized, and ethanol tolerance was enhanced and broadened by a second phase of adaptation in ethanol-challenged continuous culture. While exposure to only PSGHL was also sufficient to cause significant improvement of the performance of *S. stipitis* in enzyme hydrolyzates of both AFEX-pretreated corn stover and dilute acid-pretreated switchgrass, the exposure to AFEX CSH and/or ethanol-challenged continuous culture followed by PSGHL did not appear to generate additional performance advantages over the simple exposure to one hydrolyzate or the other. The reverse order of PSGHL exposure followed by AFEX CSH was not investigated, however. Preferred hydrolyzate applications for the top isolates are summarized in Table 2.

The competitive advantages of adapted strains were strongly related to significant improvements in sugar uptake rates and especially xylose uptake rate. Improvements in sugar uptake rate translated to increased ethanol productivity, particularly on xylose. However, adapted strains often demonstrated only modest improvements over the parent strain in maximal ethanol accumulation and yield per initial sugar. Based on the results of studies in a defined medium optimized for *S. stipitis* (ODM), the mechanisms of improved hydrolyzate performance varied among strains. Most strains, except those exposed to both AFEX CSH and PSGHL types of hydrolyzate, demonstrated reduced diauxic lag during transition from use of glucose to xylose. All strains showed better xylose fermentation performance in the presence of acetic acid, but relative performance depended on the level of prior induction on xylose. One strain (2A.1.30R2-E40-C5) showed significantly better growth resilience to acetic acid in the 6 to 15 g/L range on xylose, and the same strain showed shorter lag time in the presence of 25 mM furfural. However, in general, low cell densities of adapted strains were as susceptible to furfural-induced growth lag as the parent strain.

The incorporation of a variety of nutrient supplements into high solids loading hydrolyzates, including low cost commercial sources, was key to controlling the dynamic range of isolate performances for improved statistical separation during screening. The importance of nutrients to successful fermentation of concentrated hydrolyzates is perhaps due to an elevated need for amino acids and perhaps other nutrients required for cell maintenance, redox balancing, and repair. Additionally, the variation of supplements and hydrolyzate types during the screening process allowed identification of the most robust isolates with ability to function consistently in a variety of culture environments, as might be encountered in an industrial setting. The resulting new strains of *S. stipitis* NRRL Y-7124 were characterized by a greatly reduced growth lag and economically harvestable accumulations of ethanol at faster rates than previously reported for non-genetically engineered yeast fermenting hydrolyzates at pH 5 to 6 without prior overliming or other detoxification measures. The evolved yeast strains will support lower cost production of renewable ethanol from agricultural biomass, reducing dependence on fossil fuels.

Our studies so far provide a valuable set of *S. stipitis* isolates related by evolution under different selection pressures and an associated catalog of their kinetic attributes pertinent to hydrolyzate fermentation to ethanol biofuel. Future studies utilizing genome sequencing tools are expected to reveal how the genetic structure of the various superior isolates (Table 2) has changed from one phase of evolution to the next, following the flow diagram of selection pressure phases traversed by the evolving *S. stipitis* population in Figure 1. This figure essentially provides a useful road map notating which of the top isolates (Table 2) were obtained from each phase, such that it may be possible to associate adaptation method with specific genetic changes obtained. Fermentation performance characteristics indicated in the figures and tables may also be logically associated with the genetic changes that have occurred in each isolate. Such future work promises valuable new knowledge to help with the continued research and engineering of next-generation yeast biocatalysts and processes for converting lignocellulosic biomass to biofuels.

## Methods

### Stock cultures

A lyophilized culture of the parent strain *Scheffersomyces stipitis* NRRL Y-7124 (CBS 5773) was acquired from the ARS Culture Collection (National Center for Agricultural Utilization Research, Peoria, IL). Stock cultures of the strain NRRL Y-7124 and its derivatives were maintained in 10% glycerol at −80°C. Glycerol stocks were used to inoculate yeast malt peptone dextrose (YM) agar plates which were incubated 48 to 72 h at 25°C [11]. Developed plates were stored up to a week at 4°C prior to use as liquid preculture inocula.

### Optimal defined medium (ODM)

The following defined medium composition optimized for ethanol production from high xylose concentration feeds [10] was used in all precultures and growth cultures for inhibitor tolerance bioassays: *purines/pyrimidines* - 10 mg/L adenine, cytosine, guanine, uracil, and thymine; *macro-minerals* - 1 g/L K_2_HPO_4_, 1 g/L KH_2_PO_4_, and 0.75 g/L MgSO_4_^**.**^ 7H_2_O; *trace minerals* - 10 mg/L NaCl, 50 mg/L FeSO_4_^**.**^ 7H_2_O, 5.5 mg/L ZnSO_4_^**.**^ 7H_2_O, 1.6 mg/L CoCl_2_^**.**^ 6H_2_O, 12.5 mg/L MnCl_2_, 5 mg/L (NH_4_)_6_(Mo_7_O_24_) ^**.**^ 4H_2_O, 8 mg/L CuSO_4_^**.**^ 5H_2_0, 27.5 mg/L CaCl_2_ H_2_O, 250 mg/L EDTA; *vitamins* - 0.5 mg/L biotin, 0.5 mg/L thiamin, riboflavin, calcium pantothenate, niacin, pyridoxamine, thioctic acid, 0.05 mg/L folic acid, and B_12_; *carbon and nitrogen sources* - the medium was originally optimized to accommodate efficient conversion of 150 g/L xylose with 0.15 *M* N supplied 80% by 3.56 g/L urea and 20% by amino acids (10 g/L Difco Vitamin-Assay Casamino acids (Difco, Corpus Christi, TX, USA) (product 228830) + 0.1 g/L D,L-tryptophan (Sigma-Aldrich T3300, Sigma-Aldrich, St. Louis, MO, USA) + 0.4 g/L L cysteine (Sigma-Aldrich C7352)) [10]. In certain instances as designated, it was applied at one-third of the sugar and nitrogen source loadings - that is, with 50 g/L xylose or glucose and 0.05 *M* N as amino acids and/or urea to maintain C:N at 33:1, which is near the optimal 37:1 ratio.

### Hydrolyzates

The comparative compositions of AFEX CSH at 6% and 12% glucan levels, PSGHL, and the enzyme saccharified switchgrass hydrolyzate (SGH) are given in Table 1. Hydrolyzate preparation details are given below. To study the impact of nutrient supplementation, SGH was amended with the following nutrients, then filer sterilized for use in isolate performance screening: SGH-N1(nutrient level 1) = SGH + 6.66 g/L casamino acids, 0.066 g/L tryptophan, 0.266 g/L cysteine + 2.36 g/L urea + ½ of liquid vitamin stock for ODM + ½ of dry MgSO_4_ for ODM added prior to pH adjustment to 5.6 +/− 0.1 followed by filter sterilization of the finished hydrolyzate. SGH-N2 (nutrient level 2) = SGH supplemented with soy flour (ADM Toasted Nutrisoy Flour, Product Code 063160) and then 2.36 g/L urea to yield the nutrient levels set forth in Table 2, and pH was adjusted to 5.75. This mixture of soy and urea nitrogen sources provided the optimal approximately 20% primary amino nitrogen and approximately 80% nitrogen as urea noted previously for *S. stipitis* NRRL Y-7124 when the ODM sugar loading was approximately 100 g/L sugars [10].

#### Preparation of AFEX CSH at 6% and 12% glucan

Corn stover harvested in September 2008 was obtained from Arlington Research Station located in WI, USA. The corn hybrid used in this study was Pioneer 36H56 (triple stack - corn borer/rootworm/Roundup Ready (Monsanto, Creve Coeur, MO, USA)) variety. The biomass size reduction was done first by using hammer mills and dried at room temperature until the moisture content of the biomass was <10% (dry weight basis). Then, further fine milling was done using a Thomas Model 4 Wiley® Mill (Swedesboro, NJ, USA) to 4 mesh size (0.5 cm) and stored at 4°C in zip-lock bags until further use. AFEX pretreatment was carried out using a 5-gallon high pressure stainless steel batch reactor purchased from Parr Company (Moline, IL, USA) at Michigan Biotechnology Institute (MBI) pretreatment facility (Lansing, MI, USA) [14]. About 750 g of biomass was pretreated in a batch process. Biomass moisture content was raised to 60% by spraying de-ionized water and loaded into the reactor. The reactor was then charged with nitrogen followed by pumping of anhydrous liquid ammonia using an ammonia delivery system (comprised of an ammonia pump and a flow meter) into the reactor at 1:1 ammonia to biomass ratio. The reactor was then heated up using a heating mantle until the temperature of the biomass reached 100°C (approximately 300 psi). This condition was maintained for a period of 30 min, and the ammonia was released by venting. The pretreated biomass was then transferred to a plastic tray and dried in the hood overnight to remove residual ammonia present in the biomass. The AFEX-treated biomass was then packed in a plastic zip-lock bag and stored at 4°C until further use.

AFEX-pretreated biomass was hydrolyzed at high solids loading (6% and 12% glucan loading) using commercial enzymes supplied by Novozymes (Franklinton, NC, USA) and Genencor (Palo Alto, CA, USA) at 30 mg/g of glucan enzyme loading (70% Ctec2, 15% Htec2, and 15% Multifect Pectinase). Enzyme hydrolysis was done under sterile conditions using 2-L baffled shake flasks at 50°C, 250 rpm for 168 h. The pH was maintained at 4.8 using 3 M HCL. Biomass was loaded in two (6%) to three (12%) batches during hydrolysis to overcome mixing problems caused due to high viscosity during the initial stages of hydrolysis [15]. After the completion of hydrolysis, the hydrolyzate slurry was transferred to 1-L centrifuge tubes and spun at 6,000 rpm for 30 min to remove the solids from the liquid using Beckman Avanti centrifuge system (Brea, CA, USA). The hydrolyzed sugar stream (supernatant liquid) was sterile filtered using a 0.2 μm Millipore (Billerica, MA, USA) sterile-cup membrane filtration system and stored at 4°C until further use.

#### Preparation of PSGHL

Switchgrass hydrolyzates were prepared from Kanlow N1 baled post-frost from Mead, NE, USA, that was milled to pass through a 2-mm screen. Switchgrass was pretreated at the 20% solids level by mixing 20 g dry weight of biomass with 80 mL of 0.936% (v/v) sulfuric acid solution and 0.3 g Pluronic F-268. Each of 12 closed stainless steel vessels was loaded with reactants and mounted in a Mathis Labomat IR Dyer Oven where they were rotated at 50 rpm (1 min right then 1 min left) and heated to 160°C, held for 15 min, and then cooled at 40°C. To prepare PSGHL, the pretreatment reaction products were centrifuged for 45 min at 7,000 rpm and sterile filtered through 0.2 μm Nalgene filter units. Supernates were combined and adjusted with Ca(OH)_2_ to pH 6.0 to 6.5, and the resulting PSGHL was filter sterilized and refrigerated at approximately 4°C until use.

#### Preparation of SGH

To prepare SGH, switchgrass was pretreated in the Labomat oven at the 20% solids level as described above. After pretreatment, the product was adjusted to pH 4.5 by adding 7.14 mL of 15% Ca(OH)_2_ solution and 4.5 mL of 1 M citric acid buffer directly into each vessel and then tumbling 15 min in the Labomat. Pretreatment hydrolyzates were transferred to 250 mL pyrex bottles for saccharification. To each bottle, 2.7 mL of CTec and 0.5 mL of HTec enzymes (Novozyme) were added. Tightly capped bottles were incubated approximately 72 h at 50°C and 175 rpm. Resulting hydrolyzates were sterile filtered through 0.2 μm Nalgene filter units and refrigerated at 4°C until use.

### AFEX CSH serial transfer culture adaptation process

A preculture of strain NRRL Y-7124 was inoculated by loop transfer of cells from YM agar to 75 mL ODM + 150 g/L xylose to challenge growth under osmotic stress. Precultures in 125-mL flasks with Bellco silicon sponge closures (Bellco, Vineland, NJ, USA) were incubated 24 h at 25°C with shaking (150 rpm, 1″ orbit). Frozen aliquots of 6% to 12% glucan AFEX-pretreated corn stover hydrolyzate were thawed in cold water and used at pH 5 to prepare a dilution series in 96-well microplates. Plates were filled with 50 μL per well and eight wells per dilution, then inoculated with a few microliters of preculture per well to allow for an initial absorbance (620 nm) A_620.0_ ≥ 0.1. Plates were statically incubated in a plastic box with a wet Wypall for humidity at 25°C for 24 to 48 h. Using the most concentrated hydrolyzate dilution that visibly grew (that is, A_620_ > 1), 1 to 5 μL was transferred to each well of a new hydrolyzate dilution series (A_620.0_ ≥ 0.1). Cell growth was monitored by culture absorbance (620 nm) using a plate-reading spectrophotometer (Biotek PowerWave XS; Biotek, Winooski, VT, USA). An uninoculated dilution series served as a control and blank. Glycerol stocks of adaptation cultures were prepared regularly for subsequent isolation of improved strains or use in reinoculating continuing hydrolyzate dilution series by mixing 200 μL of the greatest hydrolyzate concentration colonized with an appropriate glycerol solution to suspend cells in 10% glycerol in cryovials for freezing at −80°C.

For isolation of single tolerant colonists, selected glycerol stocks of adaptation cultures were streaked to YM agar and used to inoculate 50 μL of 3% glucan hydrolyzate (pH 5) in each of three microplate wells to A_620,0_ = 0.1. The 96-well microplates were incubated as before at 24 h and 25°C. Colonized culture wells were pooled and dilution plated to YM agar or 6% glucan AFEX CSH agar. Selected single colonies were picked from the highest dilution plated after 24 to 48 h incubation at 25°C and then restreaked to YM plates for incubation 24 h prior to glycerol stock preparation.

#### Batch and fed-batch culture evaluations of AFEX CSH utilization

For evaluation in 6% glucan AFEX CSH batch cultures, cells from 48-h plates streaked from glycerol stocks were suspended in buffer to A_620_ = 10 and 1 μL used to inoculate each of four wells of 50 μL 3% glucan hydrolyzate (12% glucan AFEX CSH at pH 5 diluted 1:3 with sterile water) to A_620_ = 0.2. Microplates were developed 24 h and then two wells were transferred to inoculate precultures of 25 mL of pH 5 6% glucan AFEX CSH/50-mL flasks with Bellco silicon sponge closures. The precultures were incubated for 24 h at 25°C, approximately 150 rpm (1″ orbit) and then used to inoculate similar 25-mL growth cultures to A_620_ = 0.1. The cultures were incubated as for precultures and sampled daily (0.2 mL) for monitoring biomass accumulation (A_620_) and concentrations of sugars and fermentation products (HPLC).

The performance of AFEX 6% glucan CSH-grown populations repitched to 8% glucan hydrolyzates was also studied. Inocula for 30-mL growth cultures were prepared as described above for AFEX 6% glucan CSH batch cultures. Growth cultures were inoculated to an absorbance of 0.1 and incubated in 50-mL flasks with silicon sponge closures (Bellco). Cultures were sampled daily for A_620_ and HPLC analyses. When the majority of the xylose had been consumed, the cells from growth cultures were harvested by centrifugation and repitched to A_620_ of 40 in 4.2 mL of 8% glucan AFEX CSH in 50-mL flasks with septum caps, vented with 3/8″ 26 G needles. All cultures were incubated at initial pH 5, 25°C, 150 rpm, 1″ orbit. The daily samples were plated for viable cells, A_620_ was measured, and the remaining sample was centrifuged to collect supernate for HPLC analyses.

In later studies, repitched AFEX 6% glucan CSH cultures were fed with 12% glucan hydrolyzate. Following the above procedure, cells were harvested by centrifugation and resuspended to an A_620_ of 50 in 4.2 mL of 6% glucan AFEX CSH (pH 5) in a 50-mL flask with vented septum cap. All flasks were incubated as before. After approximately 24 h and significant sugar consumption in the 6% glucan hydrolyzate, the cultures were then fed with 4 mL of 12% glucan hydrolyzate (pH 5). The cultures were sampled prior to the feed and thereafter.

#### Evaluation of diauxic lag on ODM with mixed sugars and tolerance of acetic acid

Precultures of 75 mL ODM with 150 g/L xylose in 125-mL flasks with silicone sponge closures (Bellco) were inoculated by loop transfer from YM glycerol streaks. The 24-h precultures were used to inoculate similar 75-mL test cultures but with ODM containing 75 g/L of glucose and 75 g/L of xylose. All flask cultures had initial pH 6.5 and were shaken at 25°C, 150 rpm (1″ orbit). The impact of 0 to 15 g/L acetic acid on strain fermentation of glucose and xylose was also tested using a similar protocol except that the initial pH was set at 6.0 ± 0.2 in test cultures to buffer the impact of acetic acid consumption.

### Continuous culture selection for ethanol-challenged xylose utilization

The continuous culture feed medium was ODM with 60 to 100 g/L xylose, 20 to 50 g/L ethanol at pH 6.3 ± 0.2. AFEX-tolerant colony 5 was precultured in 75 mL of the ethanol-free feed medium in 125-mL flasks at 25°C, 150 rpm (1″ orbit) for 24 h. The continuous culture was initiated with colony 5 preculture by inoculating 100 mL of ODM + 100 g/L xylose + 20 g/L ethanol to A_620.0_ approximately 0.5. The 100-mL culture holding volume was maintained at 25°C in a jacketed 100-mL spinner flask (Bellco) stirred at 200 rpm and outfitted with a sterilizable pH electrode. Temperature was controlled with a refrigerated circulating water bath. For the first 125 days of cultivation, the feed medium was dosed using a pH actuated pump such that when the culture fermentation was sufficient to drop the pH to 5.4, the feed medium at 100 g/L xylose and 50 g/L ethanol would dose to prevent the pH from dropping lower. A continuously pumping effluent pump drawing from the culture surface maintained the fermentor volume. Thus, the ethanol concentration of the culture rose at an artificially high rate in response to fermentation progress. Samples (1 to 2 mL) were taken from continuous cultures every 48 to 72 h and analyzed for A_620_, cell viability, sugars, and ethanol. Effluent was collected and measured at sample times. Based on effluent collection rates, the dilution rate varied from 0 to 0.01 h^−1^. Glycerol stocks were saved on a regular basis by isolating from viability spread plates allowing formation of 30 to 100 colonies, which would be a sampling of the most prevalent, robust colonists at that point in the enrichment process. This plate was flooded with approximately 5 mL of 10% glycerol to prepare duplicate cryovials. On occasion, it was necessary to restart the continuous culture using the most recent glycerol stock.

Once pH-actuated continuous fed cultures were able to grow solely on xylose in the presence of up to 28 g/L ethanol, the remainder of the continuous culture selection process (next 80 days) was carried out at a dilution rate of approximately 0.012 h^−1^ using ODM + 60 g/L xylose + 30 to 50 g/L ethanol. The selection culture was restarted from the current, most resistant glycerol stock population streaked to YM and transferred to a preculture of ethanol-free ODM with 60 g/L xylose for incubation as above. The 100-mL holding volume of ODM with 60 g/L xylose and 20 g/L ethanol was inoculated to A_620_ 0.5, and the population was allowed to grow batch-wise to stationary phase; then the feed medium flow was started. Over time, ethanol concentration in the feed was raised as yeast tolerance improved and advancing populations were captured in glycerol stocks for use as described above.

During the last 6 months of operation, ultraviolet (UV) irradiation was used approximately monthly to induce further mutations in the glycerol stock populations used to restart continuous cultures which were fed as before. Colonies from plates streaked with glycerol stocks were resuspended in 10 mL of ODM with 60 g/L xylose (as used for precultures) and transferred to a common sterile flask. The combined cell suspension at approximately 5 × 10^8^ viable cells/mL was used to cover the bottom of four to five petri plates. Each open plate was placed below the UV light source in a biological safety cabinet and exposed for 45 min. The excess cell suspension remaining after filling plates and a post-irradiation sample were dilution plated to allow estimating the kill rate at approximately 97%. The UV-exposed cultures (approximately 30 mL) were transferred to a foil-covered 50-mL flask to prevent photo reactivations and to preserve mutations as cultures were incubated at 25°C and 150 rpm for 24 h while viable cell counts returned to approximately 1 × 10^8^ viable cells/mL for continuous culture inoculation to approximately 1 × 10^7^ viable cells/mL.

#### Performance of adapted population growth and fermentation of xylose with ethanol present

In order to focus isolation efforts, selected glycerol stock cultures were screened to identify those with best growth and fermentation of xylose in the presence of ethanol. First, xylose uptake by glucose-grown high cell densities was evaluated in the presence of ethanol. Precultures in ODM with 150 g/L glucose were incubated as described above for 96 h prior to use as inocula for the test flask cultures in order to produce large populations requiring enzyme induction for xylose utilization. Test cultures in ODM +60 g/L xylose + 30 to 45 g/L ethanol were inoculated to A_620_ of 40 and incubated at 25°C, 150 rpm (1″ orbit) in 50-mL flasks with silicon sponge closures.

Growth on xylose in the presence of ethanol was also evaluated. Precultures were inoculated by loop transfer to 75 mL of ODM with 150 g/L xylose in 125 mL flasks and incubated as previously described. Test cultures were inoculated to an A_620_ of 0.1 in 25 mL of ODM + 60 g/L xylose + 30 to 45 g/L ethanol in 125-mL flasks. Flasks were incubated at 25°C, 300 rpm, 1″ orbit, and sampled.

#### Isolation of single-cell colonies utilizing xylose in the presence of ethanol

For each glycerol stock showing superior ability to grow on and ferment xylose in the presence of ethanol, 1-mL precultures on PSGHL mixed 1:1 with ODM + 50 g/L xylose (no ethanol) were inoculated by picking from glycerol stock streaks. Precultures were contained in 96-well, deep-well plates with low evaporation covers (Duetz clamping system, Applikon Biotechnology, Delft, The Netherlands) and incubated 48 h at 25°C, 400 rpm, 1″ orbit. Precultures were used to inoculate 16 × 1 mL replicate cultures to A_620_ = 0.5 in 1:1 PSGHL:ODM + 50 g/L xylose with 20, 30, or 40 g/L ethanol for enrichment of tolerant colonists. Enrichment cultures were incubated similarly to precultures. Harvesting from highest ethanol concentration allowing growth and xylose use, each cell line was plated to YM agar to obtain single colonies. Ten colonies per cell line were picked and streaked to new YM agar plates for glycerol stock preparation.

### PSGHL serial transfer culture adaptation process

Precultures of NRRL Y-7124 (parent), 5MSU colony 5, and 2A.1.53R in 75 mL of ODM + 150 g/L xylose were prepared and incubated as described above for the AFEX CSH hydrolyzate adaptation process. PSGHL was diluted with water to provide a series of increasing concentrations in 96-well microplates for each of the three cell lines. Each 50 μL micro-culture of the dilution series was initiated to A_620_ ~ 0.1 to 0.5 with precultures. The PSGHL adaptation was carried out using the same procedure as the AFEX CSH hydrolyzate adaptation detailed above, and glycerol stocks of progressive populations were prepared. Single-cell isolates were also obtained directly from final (480 day) adaptation plates by dilution plating each of the three cell lines to YM agar since all were growing in the full strength PSGHL. Ten large colonies were picked for each of the three cell lines and then bar streaked to YM plates for glycerol stocks.

Glycerol stocks of the three cell lines at two earlier points of adaptation (360 and 420 days) were streaked to YM agar, precultured 24 h on ODM + 50 g/L xylose, then challenged in 50 μL of 50% PSGHL per microplate well incubated statically 48 h from initial A_620_ ~ 0.2, then spread onto PSGHL gradient plates ranging from 0% to 50% strength hydrolyzate (delivering approximately 300 to 400 viable cells per plate), and ten single colonies picked per cell line from the highest hydrolyzate concentration area of the gradient [29]. Picked colonies were streaked to YM for glycerol stock preparation. Alternatively, cell lines were propagated from glycerol stocks as for selective plating on gradient agar, but instead of the gradient agar plates, 96-well microplates with a range of hydrolyzate concentrations from 50% to 100% strength and ethanol from 10 to 40 g/L were inoculated to A_620_ ~ 0.2. Microplates were developed 72 to 96 h with the following conditions: 50 μL/well, static incubation, 25°C, humidified box. Via dilution plating on YM agar, ten single colonies were isolated from wells of the harshest hydrolyzate-ethanol combinations showing growth, and glycerol stocks were prepared as above.

### Performance ranking screens to select best isolates from AFEX CSH, ethanol, and dilute acid SGH adaptation phases

#### Deep-well plate screen of PSGHL performance as primary elimination of inferior isolates

Five sets of thirty isolates were screened along with NRRL Y-7124 parent (control) to choose six top strains from each set of 30. For higher throughput, the screen was carried out in Applikon Duetz System deep-well plates filled with 1 mL per well and covered with stainless steel lids with black silicone low evaporative seals. Plates were incubated in a New Brunswick Innova 42R shaker (Eppendorf, Hamburg, Germany) at 25°C and 400 rpm (1″ shaker orbit). Isolates were picked from glycerol streaks to duplicate wells of ODM + 50 g/L xylose and incubated 48 h. All deep-well plate filling patterns were designed to allow separation of different isolates by open wells. The 50% PSGHL was prepared by mixing PSGHL 1:1 with ODM + 10 g/L glucose + 50 g/L xylose. A 50-μL volume of ODM precultures (A_620_@ ~ 10) was transferred to each of the two 50% PSGHL wells for each of the isolates and controls to obtain an A_620_ of approximately 0.5. Cells of each isolate were harvested at 72 h from the 50% PSGHL challenge precultures and used to inoculate 50 μL per 1,000 μL to A_620_ ~ 0.5 in five deep wells for each of two test media: 60% PSGHL + ODM nutrients and 75% PSGHL + ODM + YM nutrients. In the two test media using the indicated partial strength of PSGHL, the nutrients (excluding sugars) were at half of the strength as standard for ODM (when designed for use with 50 g/L sugar). When present, YM nutrients were also used at half of the standard strength of 3 g/L yeast extract, 3 g/L malt extract, and 5 g/L peptone. For each sampling, a well was transferred to centrifuge tubes to obtain supernate (7,000 rpm, 15 min) for ethanol, glucose, and xylose high throughput analyses. Biomass was measured in 96-well plates (200 μL/well) with a Biotek Powerwave spectrophotometer. Within each of the five sets of isolates tested, relative performance indexes were calculated and used to rank each strain based on ethanol yield and xylose uptake rate on both test media.

#### Comparison of top PSGHL performers on SGH at different nutrient levels

The top 32 isolates performing in the deep-well plate screen of PSGHL and the parent strain NRRL Y-7124 control were next screened twice in 16-mL flask cultures on SGH and SGH amended with two levels of nitrogen, SGH-N1, and SGH-N2. Isolates were picked from glycerol streaks to duplicate deep wells of 1 mL ODM + 50 g/L xylose as before and incubated 48 h in the Duetz System. Then, 50 μL of ODM precultures was transferred to 50% SGH challenge cultures which were incubated in the Duetz System. The 50% SGH was prepared by mixing SGH 1:1 with sugarless ODM + 50 g/L xylose (pH 5.6). Isolates were harvested after 72 h from the 50% SGH challenge precultures (A_620_ ~ 10). For each of the isolates, a 16-mL aliquot of SGH, SGH-N1, or SGH-N2 was inoculated with the cell pellet (15 min, 4,900 rpm) from three wells of challenge culture to yield initial test culture A_620_ ~ 2.0. Test cultures were incubated at 25°C, 180 rpm (1″ orbit) in 25-mL flasks with Bellco silicone sponge closures. Flasks were sampled, and samples were analyzed per the PSGHL screen.

#### Comparison of top SGH performers on AFEX CSH

The top 21 isolates performing in the deep-well plate screen of SGH and the parent strain NRRL Y-7124 control were next similarly screened twice in 16 mL flask cultures on 6% glucan AFEX CSH at pH 5.2.

### Comparative kinetics of superior isolates

Diauxy during glucose and xylose fermentation at low cell density (A_620.0_ 0.1) on ODM was evaluated as previously described for AFEX CSH isolates. Additionally, the impact of acetic acid on fermentation of ODM with mixed sugars and diauxy at high cell density was also evaluated. Precultures were inoculated by loop transfer from YM glycerol streaks to 200 mL ODM with 150 g/L xylose or glucose at pH 6 in 300-mL flasks with silicone sponge caps (Bellco). Flasks were incubated at 150 rpm (1″ orbit) for 96 h at 25°C. Test cultures were inoculated to an A_620_ of 50 using cell pellets from precultures in 12 mL ODM + 75 g/L xylose with 0 to 15 g/L acetic acid at pH 6.0. The 12-mL treatments were distributed 1 mL per well to 12 wells of a deep-well plate and incubated in the Duetz System.

To study impact of inhibitors on growth, liquid precultures were inoculated by sterile loop in 20-mL cultures of ODM with 50 g/L xylose (or glucose) at pH 6.0 in 50-mL flasks with silicone sponge caps. Flasks were incubated at 150 rpm (1″ orbit) for 24 h at 25°C. Test cultures were inoculated to an A_620_ of 0.1 in duplicate wells containing 0.8-mL volumes of ODM + 50 g/L xylose (or glucose) +/− acetic acid or furfural inhibitors at pH 6.0 in 48-well MTP flower plates (P/N MTP-48-BOHS, M2P Labs, Baesweiler, Germany). Test cultures were incubated at 25°C, 1,100 rpm in a Biolector Instrument where light scattering was monitored.

For larger-scale hydrolyzate fermentations in flasks for comparative kinetics, liquid precultures were inoculated by sterile loop to 75-mL cultures of ODM + 50 g/L xylose in 125-mL flasks (silicone sponge caps) and incubated 48 h, 150 rpm (1″ orbit), 25°C. The 48-h precultures were used to inoculate 75-mL challenge cultures of 1:1 SGH-N2:water (pH 6.2) to A_620_ ~ 0.5, and they were incubated similarly in 125-mL flasks. Challenge cultures were harvested at approximately 72 to 96 h as required to obtain populations in the midst of xylose consumption. Test cultures were inoculated at A_620_ 8.4 ± 2.5 to 75 mL SGH-N2 (pH 6.2) incubated in 125-mL flasks with silicone sponge caps at 25°C, 150 rpm (1″ orbit). For low initial cell density experiments, test cultures were inoculated to A_620_ 0.5 in 23 mL SGH-N2 per 50-mL flask.

### Analyses

Cell biomass was measured by culture absorbance at 620 nm in 1-cm cuvettes using a Genesys 2 spectrophotometer (0.167 g/L biomass per unit absorbance) or in microplates using a BioTek Powerwave XS plate reader (200 μL/well). All absorbance data were reported in terms of Genesys 2 absorbance units (equivalent to microplate absorbances/0.438). Quantitations of sugars, ethanol, furfural, HMF, and acetic acid in culture samples were by Waters HPLC system equipped with a Biorad HPX-87H Aminex ion exclusion column (Bio-Rad, Hercules, CA, USA) fitted with a Micro-guard Cation H Micro-Guard Cartridge (125–0129) precolumn. Samples (10 μL) injected to the precolumn were isocratically eluted at 60°C with acidified water (15 mM HNO_3_) at 0.6 mL/min to achieve component separations which were monitored by both refractive index and ultraviolet absorbance detectors (215 nm). For hydrolyzate compositional analysis, Biorad Aminex HPX-87P carbohydrate analysis column (125–0098) (with Deashing cartridge (125–0118) and Carbo-P Micro-Guard Cartridge (125–0119)) was used at 80°C with water mobile phase. For higher throughput analyses of isolate screenings in deep-well micro-plates, a Biorad Fast Acid Analysis column (125–0100) at 0.6 mL/min acidified water mobile phase was used to assay ethanol, while glucose and xylose were analyzed in microplates using a YSI 2900 Biochemistry Analyzer.

For available nitrogen, enzyme-based test kits were used to assay primary amino nitrogen, ammonia, and urea (Megazyme International Ireland Ltd., Wicklow, Ireland).

Analysis of variance (ANOVA) and Student-Newman-Keuls (SNK) pairwise comparison analyses were performed using Sigmastat 3.5 (Systat Software, Inc., San Jose, CA, USA) at significance criterion *P* ≤ 0.05.

## Abbreviations

A: spectrophotometric absorbance
AFEX: ammonia fiber expansion
AFEX CSH: AFEX-pretreated corn stover hydrolyzate
AFEX CSH > E: adaptation stress was AFEX CSH followed by ethanol
C:N: carbon to nitrogen ratio
E: ethanol
F: statistical parameter dependent on observed data, data average, and standard deviation that is used in the calculation of RPI
HMF: hydroxymethyl furfural
NRRL Y-7124: the accession number of the parent strain of *Scheffersomyces stipitis* used in evolution studies, which is deposited in the Agricultural Research Service Culture Collection at the National Center for Agricultural Utilization Research at Peoria, IL
ODM: optimal defined medium for *S. stipitis* NRRL Y-7124 ethanol production
PAN: primary amino nitrogen
PSGHL: dilute acid-pretreated switchgrass hydrolyzate liquor
RPI: relative performance index based on fermentation rate (RPI_R_) or yield (RPI_Y_)
RPI_overall_: average relative performance based on both rate and yield parameter data
SGH: enzymatically saccharified dilute acid-pretreated post-frost switchgrass
SGH-N1: SGH fortified to 42:1 C:N with urea, casamino acids, tryptophan, cysteine, and vitamins
SGH-N2: SGH fortified to 37:1 C:N with soy flour and urea
*S. stipitis*: *Scheffersomyces stipitis*
UV: ultraviolet irradiation, used where specified to help induce mutations
YM: yeast malt peptone dextrose agar
μ: specific growth rate
